# Engaging Parents and Health Care Stakeholders to Inform Development of a Behavioral Intervention Technology to Promote Pediatric Behavioral Health: Mixed Methods Study

**DOI:** 10.2196/27551

**Published:** 2021-10-05

**Authors:** Sean M O'Dell, Heidi R Fisher, Victoria Schlieder, Tracey Klinger, Rachel L Kininger, McKenna Cosottile, Stacey Cummings, Kathy DeHart

**Affiliations:** 1 Department of Psychiatry and Behavioral Health Geisinger Danville, PA United States; 2 Department of Population Health Sciences Geisinger Danville, PA United States; 3 Autism and Developmental Medicine Institute Geisinger Lewisburg, PA United States; 4 Investigator Initiated Research Operations Geisinger Danville, PA United States; 5 Department of Pediatrics Geisinger Danville, PA United States

**Keywords:** primary care, parenting, targeted prevention, behavioral intervention technology, behavioral health

## Abstract

**Background:**

Despite effective psychosocial interventions, gaps in access to care persist for youth and families in need. Behavioral intervention technologies (BITs) that apply psychosocial intervention strategies using technological features represent a modality for targeted prevention that is promising for the transformation of primary care behavioral health by empowering parents to take charge of the behavioral health care of their children. To realize the potential of BITs for parents, research is needed to understand the status quo of parental self-help and parent-provider collaboration to address behavioral health challenges and unmet parental needs that could be addressed by BITs.

**Objective:**

The aim of this study is to conduct foundational research with parents and health care stakeholders (HCS) to discover current practices and unmet needs related to common behavioral health challenges to inform the design, build, and testing of BITs to address these care gaps within a predominantly rural health system.

**Methods:**

We conducted a convergent mixed-parallel study within a large, predominantly rural health system in which the BITs will be developed and implemented. We analyzed data from parent surveys (N=385) on current practices and preferences related to behavioral health topics to be addressed in BITs along with focus group data of 48 HCS in 9 clinics regarding internal and external contextual factors contributing to unmet parental needs and current practices. By comparing and relating the findings, we formed interpretations that will inform subsequent BIT development activities.

**Results:**

Parents frequently endorsed several behavioral health topics, and several topics were relatively more or less frequently endorsed based on the child’s age. The HCS suggested that BITs may connect families with evidence-based guidance sooner and indicated that a web-based platform aligns with how parents already seek behavioral health guidance. Areas of divergence between parents and HCS were related to internalizing problems and cross-cutting issues such as parenting stress, which may be more difficult for health care HCS to detect or address because of the time constraints of routine medical visits.

**Conclusions:**

These findings provide a rich understanding of the complexity involved in meeting parents’ needs for behavioral health guidance in a primary care setting using BITs. User testing studies for BIT prototypes are needed to successfully design, build, and test effective BITs to empower parents to take charge of promoting the behavioral health of their children.

## Introduction

### High Behavioral Health Need for Youth and Intractable Gaps in Access to Care

Behavioral health problems are common among children and adolescents [1,2]. More than 13% of preschool–age children present with disruptive behavioral problems [3], and the onset of approximately half of all lifetime cases of clinically diagnosable disorders occurs by the age of 14 [4]. Short-term consequences associated with behavioral health problems include significant impact on family functioning [2,5,6] and educational achievement [3,7]. In the long term, children with behavioral health problems have a higher lifetime risk for conduct problems, antisocial behavior, early pregnancy, drug use, and school failure [7-9]. Symptoms and impairment falling below the cutoff for diagnosis or treatment also carry a significantly higher risk for psychopathology years later. This is especially concerning considering that the prevalence of subclinical cases is twice that of those reaching clinical thresholds [10,11].

Despite the increased risk for short- and long-term negative outcomes, most children who would benefit from behavioral health care do not receive services [12,13]. Barriers to service use include structural barriers, such as shortage of behavioral health care providers, particularly in rural areas, and barriers related to stigma and negative perceptions regarding mental health problems and accessing mental health services [14,15]. In the pediatric health care setting, primary care clinicians (PCCs) often do not make appropriate referrals [16], and even when referrals are placed, many families never engage with the services [17].

Furthermore, initiatives directly aimed at increasing access to services often fail to accomplish this goal. For example, despite the efficacy of school-based programs in preventing and decreasing aggressive behavior [18,19], ongoing efforts to provide services in schools are mitigated by a variety of factors including availability of trained staff [20], stakeholder attitudes about services [21], and the attendance and participation of those students who may benefit the most [22]. Similar or higher rates of behavioral health problems in rural communities [23-25] are compounded by even lesser access to and use of behavioral health services than those in urban communities [26].

### Leveraging Innovations in Service Delivery and Technology Can Help to Close Access Gaps

Behavioral intervention technologies (BITs) have emerged as an option that may expand access to individuals for whom structural and consumer-level barriers prevent engagement with traditional face-to-face (FTF) therapy and telehealth services [27]. Most adults have a mobile phone and home internet access, far outreaching the number of individuals who live in areas with accessible behavioral health care [28]. BITs have the potential to provide better access to underserved populations and eliminate distance or transportation barriers, and they are not necessarily subject to shortages of trained staff [29,30].

Most BITs for prevention and treatment of behavioral health problems in youth have included adolescents as the primary or sole users, and promising BITs exist for a range of presenting concerns, including anxiety, depression, and chronic health conditions [31-34]. BITs designed for parents may expand access and use of behavioral health further because of the potential to engage families who may not seek FTF behavioral health care because of fear of stigma or barriers of perception and those families who may be more willing to engage in BITs that are often self-directed and relatively more private [35,36]. Indeed, looking to the internet for parenting support and behavior change strategies is an emerging trend among parents [37-40].

BITs for parents have predominantly focused on translating evidence-based parent training interventions originally developed and tested through FTF implementation [41]. There are examples of BITs for parents of children with disruptive behavior concerns that have successfully been adapted from FTF implementation for web-based platforms and have shown positive outcomes [29,35,37]. Overall, parents report a high rate of interest in and satisfaction with available BITs [40,42], yet the scope and availability of existing BITs need more development to realize this potential. One notable line of research has been conducted on the *ez*Parent Program, which is a tablet-based preventive behavioral parent training intervention adapted from the Chicago Parent Program [43] tailored for youth aged 2 to 5 years in primary care settings. An advantage of the development strategy for *ez*Parent is that many of its aspects, including implementation factors, adherence, and parental perceptions of engaging with the program, have been studied [44-46]. Nevertheless, when tested in a randomized controlled trial, *ez*Parent was not more effective on child outcomes than enhanced usual care [47]. These findings suggest that BITs such as *ez*Parent may work best in primary care settings when offered along with a range of more intensive interventions tailored to salient family characteristics that influence interest and engagement.

### Realizing the Potential for BITs to Improve Targeted Prevention in Primary Care

There is a strong potential to expand the use of BITs across a wide range of developmental, behavioral, and emotional needs beyond parenting guidance for challenging behaviors [29,35,37,40,42]. Targeted prevention in the primary care setting may help to address an important care gap because PCCs routinely engage in anticipatory guidance as part of well-child visits, but it is impractical and potentially unhelpful for PCCs to discuss every relevant domain. For example, it is estimated that if PCCs addressed every relevant prevention target with every patient according to evidence-based guidelines, then it would comprise 7.4 hours of their workday [48]; however, only 52 of 2161 recommended topics for well-child visits are considered actionable [49].

The importance of targeted prevention becomes even more salient when considering that PCCs are routinely asked to increase their roles and responsibilities (eg, developmental and behavioral health screening), yet visit lengths have not changed [50]. BITs that help PCCs *do more with less* must also consider parents’ preferences for guidance to be maximally effective. Parents often want more and different types of guidance and information than are typically provided by their child’s PCC [51,52]. Schuster et al [51] found that most surveyed parents endorsed having unmet needs regarding subjects that PCCs routinely discuss, such as crying, learning, discipline, and toilet training, and many endorsed needing more information. Combs-Orme et al [53] found that even though discipline was one of the most frequently discussed topics with PCCs, this was the area in which parents had the most questions. Therefore, research to develop BITs must also carefully examine the determinants of maladaptive parenting behaviors, such as lack of information regarding typical development and behavior or lack of parenting skills that promote healthy behavioral and emotional growth for children [54-56].

Intentionally developing BITs from the outset to meet the range of needs of families and PCCs working to address behavioral health problems may help to address the limitations of extant BITs. Research on elements of effective implementation and scaling of FTF behavioral health services in primary care has robustly shown that effectiveness is influenced by contextual factors such as provider knowledge and skills about and attitudes toward behavioral health topics, motivation to change, management and leadership practices, and financial resources [57]. This has also been shown to be relevant to BITs, as clinic personnel implementing the *ez*Parent Program reported that despite supporting the program, substantial contextual barriers impeded referrals to the program because of time, workflow, and organizational factors [58].

### This Study

This paper reports the initial stages of development for a targeted prevention BIT to empower parents to take charge of their child’s behavioral health care in pediatric primary care clinics within a predominantly rural health system in the Northeast United States. Developing targeted prevention BITs is part of an overarching approach to extend the continuum of primary care behavioral health services, including integrating behavioral health–health care stakeholders (HCS) into pediatric primary care locations and improving the scope and quality of training for PCCs in behavioral health topics.

Our approach to developing these BITs is informed by the approach described by Lyon et al [59] for adapting evidence-based psychosocial interventions for implementation in naturalistic settings. We describe the findings of the *discover* phase of development to identify the needs and perspectives of stakeholders and potential barriers to usability and implementation in the targeted intervention context. The goal is for the findings of this study to identify modification targets in extant evidence-based interventions and then apply this knowledge to iterative *design and build* cycles used to redesign interventions using prototypes and stakeholder feedback in preparation for developing a polished prototype to rigorously *test* for effectiveness in naturalistic setting. This approach is compatible with recommendations to improve BIT implementation measurement in part by distinguishing between BIT development and implementation, enhancing responsiveness to stakeholder outcomes, and integrating the BIT into existing services in the implementation context [60].

Therefore, the primary objective of this study is to identify the needs and preferences of parents and HCS within the health system that the BITs will ultimately implement, as these are the 2 key stakeholder groups which the BITs are intended to serve. We obtained input from parents and HCS using different methods to maximize the depth of information from each stakeholder group. For parents, we developed and administered a survey of parent preferences to be addressed in BITs. In addition, we developed a survey of current needs and practices for handling behavioral health concerns and administered it to a market research panel of parents within the health system. We chose to conduct a series of focus group interviews with a range of HCS to allow for more flexibility and depth of explanation of the intervention context and any associated barriers and facilitators to the implementation of BITs.

## Methods

### Design and Data Analysis Plan

We used the Pragmatic Robust Implementation and Sustainability Model (PRISM) [61] framework to inform our development activities, as it is an implementation science framework that encompasses the diverse priorities of the *design* phase by expanding the conceptualization and measurement of RE-AIM (Reach, Effectiveness, Adoption, Implementation, and Maintenance) [62] implementation outcomes by explicitly including contextual factors, overarching issues, and interdependency among components of the model. Additionally, PRISM has been shown to be compatible with qualitative methods throughout the intervention development and implementation continuum [63].

**Figure 1:**
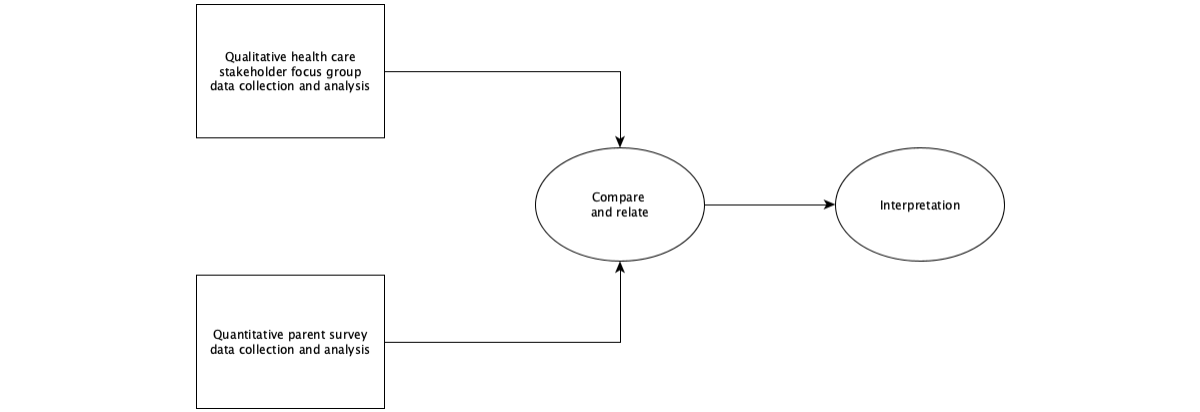
The data collection and analysis process in this study using a convergent mixed-parallel design.

We integrated quantitative survey data obtained from parents regarding their views and experiences on a variety of behavioral health topics with qualitative focus group interview data of HCS on their perceptions of unmet needs and current practices of parents regarding managing their child’s behavioral health care. To accomplish this, we employed a convergent mixed-parallel design [64] as depicted in Figure 1.

### Setting and Participants

Focus groups were conducted between April 23, 2019 and June 24, 2019 in 9 child-serving clinics within a large, predominantly rural health system. A total of 83% (48/58) of HCS participated. Participants comprised HCS from 5 primary care sites and 1 developmental medicine clinic; 2 primary care sites invited to participate declined. The primary care site focus groups were each completed in a single session in the clinic over lunch. Of these, 2 focus groups were conducted with the developmental medicine clinic stakeholders during monthly administrative meetings to accommodate the availability of participants. The focus group participants comprised a range of roles and professional backgrounds, including 16 pediatricians, 2 pediatric psychologists, 2 genetic counselors, 1 speech pathologist, 2 behavior analysts, 5 licensed nurse practitioners, 6 registered nurses, 7 physician or medical assistants, 4 patient access representatives, 1 family liaison, 1 operations manager, and 1 pediatric technician.

An electronic parent survey was conducted using Qualtrics in Spring 2019 from a geographically representative patient panel within a rural health system in the Northeast United States who had previously opted in a program to be contacted to complete web-based surveys regarding their perspectives on health care services offered within the health system in which the study was conducted. To be eligible to complete the survey, the respondent had to endorse screening items indicating that they were the parent or guardian of at least one minor child (0-18 years of age) at the time of completing the survey and were somewhat or very interested (as opposed to not at all interested) in using web-based resources to research issues and concerns they may have about their children and parenting. Invitations were distributed twice with the goal of acquiring 400 completed surveys. Of the 2240 respondents who initiated the survey, 411 met the inclusion criteria and proceeded to the rest of the survey. However, 6.3% (26/411) of these respondents abandoned the survey before completing the initial content questions. Table 1 shows the demographics of the remaining 385 respondents.

**Table 1:** Demographic characteristics of parent survey respondents (N=385).

Characteristic		Respondents, n (%)
**Age (years)**		
	18-24	6 (1.6)
	25-34	90 (23.4)
	35-44	142 (36.9)
	45-54	97 (51.2)
	55-64	38 (9.9)
	65-74	7 (1.8)
	75+	1 (0.3)
	Prefer not to say	2 (0.5)
	Missing data	2 (0.5)
**Number of children^a^**		
	1	184 (47.8)
	2	139 (36.1)
	3	40 (10.4)
	4	12 (3.1)
	5	5 (1.3)
	Missing data	5 (1.3)
**Education level**		
	High school graduate (high school diploma or equivalent including GED^b^)	37 (9.6)
	Some college	44 (11.4)
	Associate's degree in college (2-year program)	19 (4.9)
	Bachelor's degree in college (4-year program)	61 (15.8)
	Master's degree	44 (11.4)
	Doctoral degree	7 (1.8)
	Professional degree (JD^c^, MD^d^)	3 (0.8)
	Prefer not to say	4 (1)
	Missing data	116 (30.1)
**Sex**		
	Female	245 (63.6)
	Male	66 (17.1)
	Missing data	74 (19.2)
**Annual income (US $)**		
	Less than 10,000	12 (3.1)
	10,000-29,999	27 (7)
	30,000-49,999	30 (7.8)
	50,000-79,999	37 (9.6)
	80,000-99,999	26 (6.8)
	100,000 or more	58 (15.1)
	Prefer not to say	29 (7.5)
	Missing data	166 (43.1)
**Race or ethnicity**		
	American Indian or Native Alaskan	0 (0)
	Asian	4 (1)
	Black or African American	4 (1)
	Hispanic, Latino, or Spanish	4 (1)
	White	347 (90.1)
	Other	2 (0.5)
	Prefer not to say	6 (1.6)
	Missing data	18 (4.7)
**Has children in each age range (years)**		
	0 to 5	143 (37.1)
	6 to 12	143 (37.1)
	13 to 18	175 (45.5)
**Insurance type**		
	Private	44 (11.4)
	Public (Medicaid, Medicare)	35 (9.1)
	Missing data	306 (79.5)

^a^Eighteen years of age or younger.

^b^GED: General Education Development.

^c^JD: Juris Doctor.

^d^MD: Doctor of Medicine.

### Measures

#### Health Care Provider Focus Groups

The focus group moderator guide (Multimedia Appendix 1) was developed by the study authors using PRISM [61], which aims to identify and leverage multiple dimensions of internal and external contextual factors that contribute to stakeholder influence and implementation outcomes. Prompts were designed to evoke discussion among participants about the topic of unmet parental needs, including healthy development and social, emotional, and behavioral functioning of their children. The moderator introduced the study and its objectives, read prompts, and encouraged discussion among the focus group participants. Prompts also included uncovering what the HCS perceived that parents were doing to address unmet needs, and how the BIT platform website might help. In line with PRISM, participants were also asked about institutional leadership and what barriers health care HCS foresee the study team encountering in developing a mobile responsive website as a behavioral health intervention directed toward parents.

#### Parent Quantitative Survey

Questions were developed by the study authors and additional study personnel who were engaged as content experts in relevant disciplines. Given our emphasis on evidence-based content and aim to complement and expand upon the behavioral health care support provided by PCCs, content topics were selected from the American Academy of Pediatrics (AAP) anticipatory guidance recommendations described in *Bright Futures: Guidelines for Health Supervision of Infants, Children, and Adolescents* [65]. The anticipatory guidance described in *Bright Futures* covers a wide range of health, developmental, and behavioral topics across infancy, childhood, and adolescence. The study authors adopted relevant behavioral health survey topics from the *Bright Futures* topics based on their strong potential for delivery using a BIT. For example, the anxiety in children, behavioral challenges, and mood or depression in children survey topics were selected from the broader Promoting Mental Health anticipatory guidance topics from *Bright Futures*. Table 2 depicts the Bright Futures content domains and the resulting parent survey topics.

**Table 2:** Parent survey topics.

Bright Futures health promotion topics	Parent or caregiver survey topics
Promoting lifelong health for families and communities	—^a^
Promoting family support	Parenting stress; family communication
Promoting health for children and youth with special health care needs	—
Promoting healthy development	Speech or language skills, independence and activities of daily living; academic skills and intelligence; social skills; motor skills; toileting
Promoting mental health	Anxiety in children; behavioral challenges; mood or depression in children
Promoting healthy weight	Nutrition and eating
Promoting healthy nutrition	—
Promoting physical activity	—
Promoting oral health	—
Promoting healthy sexual development and sexuality	Sex and sexual development
Promoting the healthy and safe use of social media	The internet and social media
Promoting safety and injury prevention	Child safety; drugs and substance abuse

^a^Bright Futures health promotion topic not covered in parent or caregiver survey topics.

### Procedures

#### Health Care Stakeholder Focus Groups

The project manager and research assistant traveled to each clinic to conduct in person focus groups. The project manager was trained in interviewing techniques and led the focus group discussions based on the guide included in Multimedia Appendix 1. Focus groups were audio recorded and transcribed by a skilled research assistant using Start-Stop Universal software and then deidentified for analysis. Transcripts were coded by the project manager, research assistant, and 2 psychology postdoctoral fellows using Microsoft Word. The order in which the focus group interviews were coded by the study team was randomly selected using a web-based randomizing service to remove bias from the coding. An a priori codebook based on the interview guide was created to identify and code common topics within each transcript; emergent codes and themes were also identified during the course of coding. The coders individually analyzed each interview and met every week to review and establish interrater agreement. The final coded transcripts were then uploaded to Atlas.ti 8.4.15 (ATLAS.ti Scientific Software Development GmbH) for Windows, where thematic quotes could be exported into spreadsheets based on individual codes for further analysis.

#### Parent Survey

Survey respondents who met the inclusion criteria for the study rated up to 3 of the 17 topics as their top choices for content that they would be interested in learning more about through a BIT. To understand if the topic was a current challenge or if the respondent wanted more information for future reference, respondents then identified whether the topic of interest had or had not been a challenge that they had encountered so far. Next, for each of the top 3 topics, respondents were provided a list of subtopics and were asked to identify which subtopics were a problem or concern. Next, respondents were provided with a list of common strategies for addressing the broad topic (eg, anxiety in children) and asked to rate if they had used the strategies and the extent to which they perceived each strategy to be helpful. Common strategies included those with an empirical evidence base as well as those without one in the interest of learning about the prevalence and preference of a range of strategies. The survey is included in Multimedia Appendix 2.

### Data Analysis Plan

**Figure 2:**
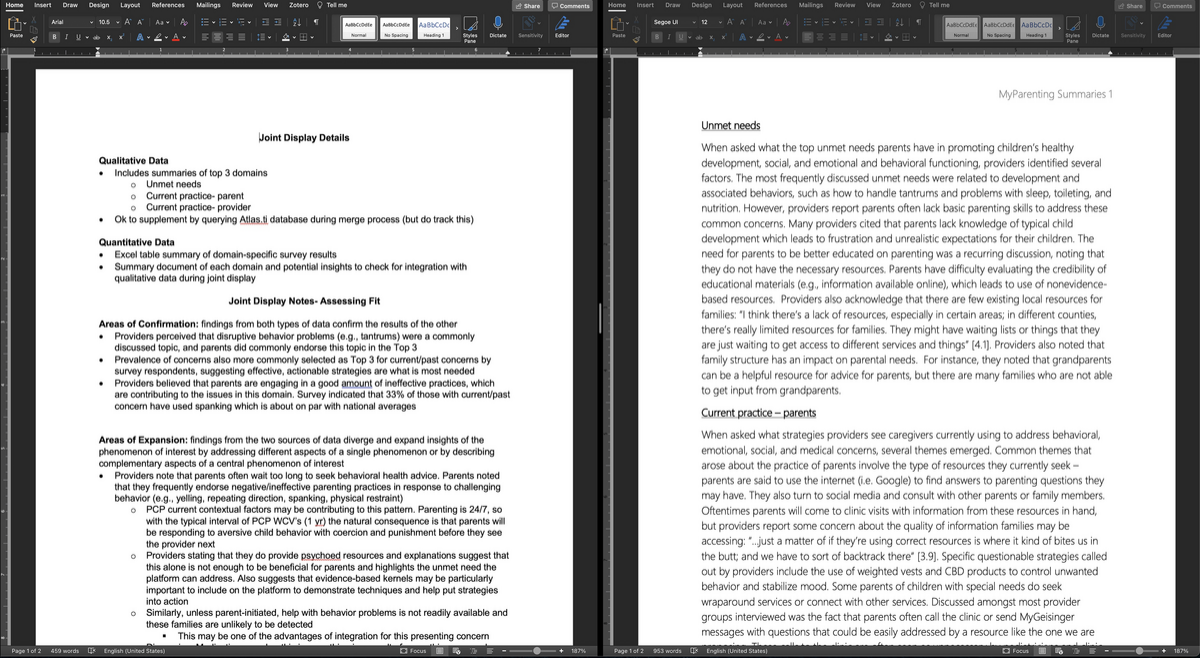
Joint display.

Parent quantitative survey data and HCS qualitative interview data were analyzed in parallel. For survey data, summaries were created using descriptive statistics for the most frequently endorsed content topics in the total sample. Descriptive statistics summarized the prevalence of endorsements for topics representing parental concerns and engagement with and perceived helpfulness of the strategies listed. Three qualitative topics from focus groups were selected for use in data integration: unmet needs, current practice providers, and current practice-parents because of their relevance to the quantitative data collected from parents. Each of these topics was analyzed and summarized by the study team based on the codes and topics identified. Each quote was then subcoded to expand on popular topics within each main code. The subcodes were utilized as framework for the overall summaries of all 3 topics. Once the parallel analyses were completed, the results were merged using a joint display to identify areas of confirmation, expansion, and discordance (Figure 2). We randomly selected 2 team members to integrate data for each survey topic, and the results were based on the comparing and relating these findings. To supplement these analyses, including those for parent survey topics that were not selected for data integration by joint display, we queried the qualitative interviews for mentions of parent survey topics and related keywords to make additional interpretations.

## Results

### Parallel Analyses of Qualitative and Quantitative Data

#### Health Care Stakeholder Focus Groups

Focus group participants reported that the most common unmet behavioral health needs of the parents they work with related to common parenting challenges such as disruptive behaviors, sleep, toileting, and nutrition. Participants commonly reported a perception that lack of foundational knowledge in promoting healthy development across behavioral health topics represented vulnerability. Other contextual factors, such as lack of easy access to credible information, were commonly reported to compound the barriers to accessing local behavioral health resources. There was also a common theme noted among participants that social networks within the family (eg, grandparents) are often resources to help with common parenting challenges.

Focus group participants noted that parents frequently turn first to web-based resources (eg, web searches and social media) to find ideas and strategies to address behavioral health needs, which often led to unproven techniques being tried first (eg, weighted vests and cannabidiol products) and, in turn, maintaining or exacerbating behavioral health problems over time. Parents’ desire for a *quick fix* was posited as an underlying reason for these choices, whereas focus group participants also noted that another subset of parents seem to follow a *wait and see* approach, in which they may wait several months to seek advice at the next routine visit, which was reported to unintentionally contribute to problems becoming ingrained and intractable. Ultimately, participants reported that this contributed to increased frustration for parents and more challenge for HCS implementing a more comprehensive and effortful course of treatment. For their part, HCS in focus group interviews reported that they made concerted efforts to spend extra time in their visits to provide guidance and psychoeducation on foundational parenting strategies. They also reported making specialty referrals when appropriate and acknowledged that they do not always have the time or resources to be responsive to parent concerns.

#### Parent Survey

Table 3 provides the frequency of each topic area selected by respondents across child age ranges of 1-5, 6-12, and 13-18 years. Across all respondents, the most frequently endorsed topics included anxiety in children (111/380, 29.2%), behavioral challenges (106/380, 27/9%), nutrition or eating (105/380, 27.6%), mood or depression in children (100/380, 26.3%), and the internet and social media (99/380, 26.1%). Responses were further examined based on whether respondents endorsed having a child of 1-5, 6-12, or 13-18 years. Anxiety in children, behavioral challenges, and nutrition and eating continued to be highly endorsed topics, regardless of child age. Respondents who reported having a child in the 6-12-year age range and the 13-18-year age range also frequently endorsed mood or depression in children and the internet and social media. Respondents who indicated they had a child aged 1-5 years also showed interest in speech or language skills, academic skills and intelligence, parenting stress, and sleep or bedtime routine.

Multimedia Appendix 3 provides descriptive statistics for the responses to each of the top 6 content topics. Most respondents endorsed each topic because of a past or current parenting challenge, as opposed to interest related to general guidance. With a few exceptions, the challenging topics listed within each topic were also endorsed by a substantial proportion of respondents, indicating that the issues parents face within each topic are often multifaceted. Similarly, respondents endorsed a variety of common strategies to help with the identified topics within each topic. Few strategies were endorsed as tried and was helpful by more than half of the respondents, suggesting that respondents are likely to try several strategies and find that few of them make a positive difference. Strategies rated as potentially problematic by the investigators are noted in the supplementary tables, and, overall, were some of the least likely strategies to be endorsed as helpful by respondents.

At the end of the survey, parents were asked if there were any additional topics they would like to see in a BIT. Of the 36 free-text responses, 7 pertained to topics that fit within the scope of the survey topics (eg, *language and speech* and *behavior*). Of the 385 respondents, 4 indicated that additional information about puberty would be helpful. Moreover, 9 responses were highly specific concerns that were outside the scope of a BIT for targeted behavioral health prevention (eg, caring for a child with a chronic illness). Several parents responded that they would have selected more or all the survey topics. Other responses pertained to coparenting, dealing with divorce, dealing with death and grief, and attachment.

**Table 3:** Parent endorsement of survey topics by age ranges of children.

Topic	Age range (years), n (%)			
	All (n=380)	1-5 (n=143)	6-12 (n=176)	13-18 (n=175)
Anxiety in children	111 (29.2)	33 (23.1)	66 (37.5)	55 (31.4)
Behavioral challenges	106 (27.9)	43 (30.1)	57 (32.4)	40 (22.9)
Nutrition and eating	105 (27.6)	51 (35.7)	44 (25)	38 (21.7)
Mood or depression in children	100 (26.3)	16 (11.2)	49 (27.8)	67 (38.3)
The internet and social media	99 (26.1)	24 (16.8)	47 (26.7)	50 (28.6)
Parenting stress	79 (20.8)	36 (25.2)	33 (18.8)	31 (17.7)
Academic skills and/or intelligence	76 (20)	28 (19.6)	40 (22.7)	35 (20)
Social skills	68 (17.9)	21 (14.7)	31 (17.6)	31 (17.7)
Family communication	63 (16.6)	19 (13.3)	28 (15.9)	34 (19.4)
Speech or language skills	45 (11.8)	29 (20.3)	14 (8)	10 (5.7)
Independence and activities of daily living	42 (11.1)	12 (8.4)	21 (11.9)	24 (13.7)
Sex and sexual development	41 (10.8)	6 (4.2)	19 (10.8)	29 (16.6)
Sleep or bedtime routine	38 (10)	28 (19.6)	13 (7.4)	3 (1.7)
Drugs and substance abuse	35 (9.2)	3 (2.1)	11 (6.3)	29 (16.6)
Child safety	28 (7.4)	22 (15.4)	13 (7.4)	3 (1.7)
Toileting	27 (7.1)	24 (16.8)	8 (4.5)	1 (0.6)
Motor skills	13 (3.4)	10 (7)	0 (0)	3 (1.7)

### Interpretations Based on Comparing and Relating Health Care Stakeholder Focus Groups and Parent Surveys

#### Overview

After parallel analyses of the qualitative health care stakeholder focus group data and the quantitative parent survey data, we compared and related features of these data to integrate and make interpretations to guide further development efforts for the BIT. The results of the data integration phase are described next according to each of the parent survey topics. Multimedia Appendix 4 presents a summary of the areas of confirmation, expansion, and discordance for selected behavioral health topics with substantial data from both sources.

#### Anxiety

Anxiety was the most prevalent concern endorsed by parents; although HCS did not identify anxiety as a pressing unmet need, there were 6 mentions of anxious or *anxiety* in the qualitative data. There was agreement between parents and HCS in the demand for more web-based resources. HCS reported that parents lack resources for behavioral health concerns, yet parents rated psychotherapy as the most helpful strategy for anxiety management. From these data, it is unclear how many families access these services. Additionally, other commonly reported parental strategies (eg, comforting the child) are not typically effective in the long term or when used as a standalone strategy, which may relate to health care stakeholder observations that parents are seeking a quick fix and need more support in long-term behavior change.

#### Behavioral Challenges

HCS and parents reported that disruptive behaviors are a common concern, but parents tend to use ineffective behavior management strategies. HCS also lacked some awareness of key parental challenges within the disruptive behavior topic. It appears that primary care provider strategies alone are not enough to be beneficial for parents. This highlights the potential benefits of a BIT to address parental needs in this area more effectively. Demonstration of specific behavior management techniques may be helpful to include in a BIT to help parents put strategies into action.

#### Nutrition and Eating

Nutrition and eating concerns were commonly reported by both HCS and parents. HCS reported that parents tended to use unhelpful strategies to manage eating concerns, but parents reported a mix of helpful and unhelpful strategies. HCS were also unaware of several common strategies parents endorsed to manage nutrition and eating concerns, and many parents indicated that they did not discuss nutrition and eating concerns with their primary care HCS. Of the queries of qualitative data returned, 3 mentions of *nutrition*, 2 mentions of *food*, and 9 mentions of *eat* were made.

#### Parenting Stress

HCS and parents both reported that parenting stress is a common concern, although parents and HCS had differing perspectives on the factors contributing to parental stress. HCS tended to discuss parental stress in terms of parental frustration with child behavior as opposed to parent-specific factors (eg, coping with emotions). The strategies parents use to manage stress may impact their use of primary care resources. Addressing parental stress is beyond the traditional scope of pediatric primary care, and HCS are likely to lack the knowledge of how to deal with more complex cases. Therefore, a BIT addressing parental stress may help HCS direct parents to useful resources.

#### Family Communication

Family communication was a commonly endorsed topic for parents, and HCS mentioned this as a concern. Furthermore, it also related to collaborative communication with HCS about child behavioral health needs. HCS perceptions of parental communication strategies were discordant with the parent-reported strategies. HCS did discuss how family structure may impact parental communication in the qualitative topics reviewed, and further queries of the qualitative data returned several mentions of *divorce*, *mixed households*, and *nontraditional families*. Interestingly, HCS expressed concern about both a lack of parental communication and excessive parental communication, whereas parents were most concerned about a lack of communication among family members. It was observed the HCS found it challenging to find common ground with parents. Similarly, parents also faced difficulty in finding common ground with other caregivers.

### Additional Interpretations of Parent Survey Topics Not Selected for Joint Display

For parent survey topics without substantial HCS qualitative data in the codes that we analyzed in the parallel phase, we searched for key terms in the qualitative data to determine if we could further compare and relate these data to make interpretations.

#### Sleep or Bedtime Routine

The parent survey topic of sleep or bedtime routine was noteworthy, in that although it was not commonly endorsed overall (38/380, 10%), it was more prevalent (28/243, 19.6%) for respondents with a child in the age range of 1-5 years. We also found 13 mentions of *sleep*, which co-occurred with our unmet needs code 7 times. More specifically, sleep patterns and sleep hygiene at different ages were brought up during at least 2 focus groups as something with which parents discussed struggling or not understanding what is normal, whether it be newborn sleep or even sleep patterns throughout childhood and adolescence. This is confirmed through coding, in that mentions of *sleep* co-occurred with the lack of knowledge code 4 times throughout the 7 focus groups.

This suggests multiple opportunities to target BIT content for young children on this topic to be most efficient with resources.

#### Mood and Depression in Children

Results regarding mood were discordant between parents and HCS. Parents commonly identified depression or mood as a top concern, but HCS did not discuss mood and depression concerns as unmet needs or in terms of strategies parents use to manage mood concerns. The qualitative data included 4 mentions of words beginning with *depress*. These mentions often co-occurred with mentions of anxiety and may suggest that HCS tend to conceptualize these as related (eg, internalizing problems) or find them frequently co-occurring in their patients. These results were somewhat surprising and may indicate a domain which improved clinical training for HCS in clinical interviewing and behavioral health screening may be helpful.

#### Drugs and Substance Abuse

Similarly, the parent survey topic of drugs and substance abuse was rated more commonly by parents with a child in the 13-18- year age range (29/175, 16.6%) than the overall prevalence (35/380, 9.2%). In reviewing the 3 mentions we found in the qualitative data of *drug*, there was poignant discussion among participants in one of the focus groups highlighting the complexity of addressing this topic with parents who are suspicious or concerned about drug abuse, how they might rely on AAP guidelines, and publications that discuss how HCS can help parents (Textbox 1).

Respondent 1 is most likely referring to the AAP clinical report by Levy et al [66]. This resource provides guidance on how pediatricians can navigate this complex and important topic for which there is presently minimal empirical literature available. Further BIT development efforts may help to design a BIT module that can provide high-quality information and resources to parents in need of guidance on this topic that they commonly reach out to their pediatrician to address.

**Exemplary quotes related to drugs and substance abuse.**

The others--the teenager who is non-compliant either at school and outperforms in other areas where they like things and how do we manage that behavior because they don't want to take away the good activities; what do I do? Or you have a parent who's suspicious of particular drug use; what do I do in this particular situation? Can we drug-test them, which is almost universal: No. However, what do we do in these situations?

Respondent 1

Why can't you do that?

Respondent 4

We can talk about that, but just ethically, no we don't do that. Ask me later...or, getting back to the drug-testing, having an explanation of, here's how to handle if you're concerned about your child's drug use, here's what you can do at home...

Respondent 1

You don’t want to know what I do at home.

Respondent 2

I guarantee he's not coming to see you.

Respondent 4

Here's the formal policy of the national organization called the American Academy of Pediatrics on how to address this with your child and our stance on drug-testing teens. It is understood that it is not just a clinic, but also nationally what is done. It would be cool to see what that does for parents.

Respondent 1

#### Parent Survey Topics With Substantive Additional Qualitative Data

Among other parent survey topics not selected for joint display, we found some useful additional information within the qualitative data that may inform future BIT development. Regarding the topic of child safety, we found that this was commonly endorsed by parents with a child in the 1-5-year age range (22/143, 15.4%); however, only 1 mention of this topic was found in the qualitative data. Speech and language skills were also commonly endorsed by parents of children in the 1-5-year age range (29/143, 20.3%), and the only mentions within the qualitative data related to accessibility of the website for parents for those who are speakers of languages other than English or may have lower educational attainment. Independence and activities of daily living were more commonly endorsed by parents of youth aged 6-12 years (21/176, 11.9%) and 13-18 years (24/175, 13.7%). The qualitative data included mentions self-help, hygiene, daily routines, and chores, which may indicate the topics that HCS most commonly discuss with parents. Finally, toileting was another topic commonly endorsed by parents of children aged 1-5 years (27/380, 7.1%); although no related mentions were found within the codes we analyzed in joint display, other qualitative data did include mentions of *toileting* (3 mentions) and *potty* (2 mentions).

#### Parent Survey Topics Without Substantive Additional Qualitative Data

The topic of academic skills and/or intelligence was commonly endorsed across age ranges (range 20%-23%), but was not selected for joint display because of a lack of discussion in the qualitative topics we included. The qualitative data also did not include terms related to child intelligence, so no more details for interpretation are available. In the focus group data, the same was true for social skills (0 mentions), the internet and social media (1 mention), sex and sexual development (0 mentions), and motor skills (0 mentions).

## Discussion

### Principal Findings

This paper on the mixed-methods study reports the initial development of a targeted prevention BIT focused on behavioral health topics for parents to be implemented in pediatric primary care within a large, predominantly rural health system. We used the *discover*, *design and build*, and *test* framework [59] to inform our development efforts. In this manuscript, we report the outcomes of the *discover* phase to gather information on the implementation context and current issues facing parents and HCS navigating behavioral health topics in pediatric primary care that a BIT can address.

Overall, the approach we selected shows promise that taking both parent and HCS input into consideration at the outset of BIT development in the *discover* phase provides unique insights that may help to address the limitations of the extant literature on BITs for parents of children with behavioral health problems. For example, research on the *ez*Parent Program, a parent-focused BIT adaptation of the Chicago Parenting Program [43], stands out among the research on BITs for parents for having carefully studied implementation and sustainability factors from the parent perspective [45,46], yet, when tested in a randomized controlled trial, it did not demonstrate superiority to enhanced usual care [47]. Findings from other research on *ez*Parent suggest that inconsistent referrals to the program were discovered only after rolling out the program in primary care and were attributed to operational workflow issues for primary care staff, and these issues were unforeseen [67]. By first studying the unmet needs of parents and HCS that a BIT might address, in the implementation context that the BIT is being developed and for the expressed purpose of extending the continuum of primary care behavioral health services already available, we may be able to obviate comparable setbacks through work in our *design* and *build* and *test* phases.

The analysis of parent and HCS data in this study provided unique insights that will help in focusing the resources on developing and conducting preliminary testing on prototypes of BITs to better meet the behavioral health needs of parents using pediatric primary care within the health system. While the extant BIT literature in this area has primarily focused on engaging adolescents with a range of behavioral health problems, including anxiety, depression, and chronic pain, in adaptations of empirically supported treatments delivered in a BIT [31-34], our results indicate that BITs for parents also have the potential to greatly expand the reach and impact of evidence-based behavioral health care. Parents reported interest in BITs across several behavioral health topics, and we learned that parent interest sometimes varied across the pediatric age range. Owing to space constraints, we highlight 1 example next. Although only 10% (38/380) of parents endorsed the sleep or bedtime routine among the top 3 concerns, twice as many parents with a child aged 1-5 years endorsed this topic (28/143, 19.6%) and relatively fewer parents of children in the 6-12-year age range (13/176, 7.4%) or 13-18-year age range (3/175, 1.7%) endorsed the topic (Table 3). The implications of such findings for resource allocation for subsequent BIT development and clinical uptake are substantial. If guided solely by the overall prevalence of endorsement, we may not have selected sleep or bedtime routine as a topic for further BIT development. By extension, knowing that 19.6% (28/143) of parents with a child between 1-5 years are interested in this topic helps us to focus our BIT development efforts on topics most relevant for this age range even though research supports the effectiveness of behavioral sleep interventions for school-age youth [68]. Insights like these deepen our understanding of more detailed feedback from parents within each behavioral health topic and help the development efforts in the *design* and *build* phase, and these may increase the likelihood of BIT uptake in clinical settings for those found to be efficacious in the *test* phase [69].

### Limitations

Our findings should be interpreted with recognition of the methodological limitations inherent to our approach, which focused on the initial development of a BIT to fit a specific implementation context. Therefore, surveys based primarily on selected *Bright Futures* topics that the research team felt would be a good fit for a BIT may not comprehensively represent the needs and preferences of parents related to empowerment to guide child development and behavioral well-being. A related limitation is that the study population is representative of the population in the region; survey respondents are mostly White and middle class; therefore, these findings may not be generalized to the needs and preferences of parents from other demographic and socioeconomic backgrounds. The sample of focus group interviewees was also recruited from the health system in which the BIT is being developed, which also introduces the possibility of limited generalizability. Finally, some caution in interpreting the findings of the data integration is warranted, given that we have not yet conducted any empirical studies to triangulate our interpretations with parent and provider interactions with BIT prototypes. Awareness of these potential limitations is also important to address in our future BIT development research because of the potential of unintentionally driving disproportionality in access to behavioral health care by developing a BIT that may not be engaging to historically excluded groups, who already face difficulties in accessing behavioral health care in rural areas [69,70]. Oversampling in the *design and* *build* and *test* phases may help in guarding against this unwanted outcome.

### Suggestions for Future Research

Our approach to the *discover* phase for the development of a BIT to empower parents to take charge of their child’s behavioral health care was shaped by our perspectives on contributing factors to the longstanding issue of limited access to high-quality behavioral health care in primary care settings. This approach may also be useful for future research developing BITs with different goals in mind. Although evidence-based treatments are often conceptualized and developed as packaged intervention products, there is usually an observed *voltage drop* when taking efficacious psychosocial treatments out of the laboratory into community practice settings [71]. This undermines the conceptualization of psychosocial treatments as a product per se, whereas conceptualization as a cocreated service between parents and HCS suggests that reduced effectiveness is not inevitable [72]. High-value behavioral health care designed with input from transdisciplinary researchers, clinicians, and patient stakeholders in the setting intended for use may provide a better chance at comparable efficacy and effectiveness [73]. The findings from our *discover* phase support the notion that usual care is a cocreated service between parents and HCS within the health system, although one which often leads to unmet needs for both stakeholder groups in the health system in which the study was conducted. Therefore, the value of a BIT can be measured against the degree to which the implementation of BITs contributed to these needs being met. Research has demonstrated that parental comfort in discussing behavioral health concerns is shaped by the quality of the PCC response; that is, when PCCs dismiss these concerns, parents report that they are less comfortable discussing these topics [74]. BITs may help in this regard, as these conversations have been shown to be brief and work well when combined with videos to illustrate effective interventions for child discipline [75].

At this juncture, we have entered the *design* *and* *build* phase to triangulate our mixed-methods findings with parent and provider feedback on the prototypes of the BIT [59]. We are currently collecting data for 2 mixed-methods user testing studies to triangulate these findings for the content topic of behavioral challenges. In 1 study, we recruited a group of parents who completed the survey and endorsed this topic in their top 3 (n=9) and another group of parents who completed the survey but did not endorse this topic in their top 3 (n=9). We chose to recruit from the parents who completed the quantitative survey to aid in triangulating findings from this study and from the behavioral challenges topic because there is substantial extant BIT literature for parents on this topic [41,47]. Another study was conducted with PCCs within the health system (n=16) to determine the usability and acceptability of provider-facing BIT to address behavioral challenges and how this can be incorporated into the electronic health record and clinic workflow.

### Conclusions

This mixed-methods study provided some unique insights into the needs and preferences of parents and HCS. These results appear useful for designing a BIT platform to enhance access to effective self-help to empower parents to take charge of their child’s behavioral health care. Future research will triangulate these mixed-methods findings with parent and health care provider reactions to BIT prototypes in preparation for an effectiveness trial on a fully functional BIT prototype.

## Appendix

Multimedia Appendix 1 Focus group moderator guide.

Multimedia Appendix 2 Parent survey.

Multimedia Appendix 3 Parent survey descriptive statistics.

Multimedia Appendix 4 Data integration summary.

## Abbreviations

AAP: American Academy of Pediatrics
BIT: behavioral intervention technology
FTF: face-to-face
HCS: health care stakeholders
PCC: primary care clinician
PRISM: Pragmatic Robust Implementation and Sustainability Model
RE-AIM: Reach, Effectiveness, Adoption, Implementation, and Maintenance
