# Supplementary material for: Engaging Parents and Health Care Stakeholders to Inform Development of a Behavioral Intervention Technology to Promote Pediatric Behavioral Health: Mixed Methods Study
Source: JMIR Pediatr Parent. 2021 Oct 5;4(4):e27551. doi: 10.2196/27551 (PMC8527378; doi:10.2196/27551)
Supplement: Multimedia Appendix 1 [file pediatrics_v4i4e27551_app1.docx]

Thank you all for agreeing to participate. My name is ­­­­­_____ and I am a qualitative researcher at ______. To start, I just want to remind you that anything you say during this focus group will remain confidential and you will not be identified in any analysis. That being said, I would like to record our conversation, if that is ok?

[Start Recorders]

If we could start with introductions, we’ll just go around the room and if you could tell me your name and title, that would be great!

“As you know, we are developing a mobile-responsive website that will host evidence-based information and strategies for parents to take charge of their children’s behavioral health care, which includes things parents can do to promote healthy development, as well as social-emotional and behavioral functioning. One reason we are developing this website is to increase parents’ access to high quality, actionable information and strategies. We also believe it is important to do what we can to ensure that the website content addresses factors that affect how parents use health care services at [Health Care System].

We’d like to get your perspective on existing interventions to improve parenting, how the quality of parenting affects aspects of the work that you do in X SERVICE LINE at [Health Care System], and what qualities of an intervention like the one we’re developing would make it useful to you and the families you serve.”

1. Program
   1. Organizational perspective (includes leadership, management, and staff)

**Topic 1: What is the unmet need?**

- **In your experience, what are the top unmet needs parents have in promoting their children’s healthy development, social-emotional, and behavioral functioning?**

Follow-up details if not covered in first question:

- - What is your sense of how unmet parenting needs are increasing complexity of care and/or cost in your service line?

**Topic 2: How do parents deal with the problem?**

- **What are parents currently doing to address these unmet needs?**
- **Probe if not mentioned: Do they seek out and use self-help resources?**

Follow-up details if not covered in first question:

- - What do you wish parents better understood or took more effective action on before they came into your office to ask for help?

**Topic 3: How can our solution be superior?**

- **What would make our website a resource that parents in your service line would use? What would take away from the website’s usability?**

Follow-up details if not covered in first question:

- - How important is it to you that you can test out the website before using it in your practice?
  - How important is it to you that you can observe results from parents’ use of the website before using it in your practice?

*“Now I’d like to ask you about some factors at [Health Care System] that are likely to affect the success of our intervention to improve parenting behaviors and promote child wellbeing”*

1. Recipients
   1. Organizational characteristics (includes leadership, management, and staff)

- **What aspects of the [Health Care System] culture support such an approach to improving parenting? What aspects may serve as barriers?**
- **To what extent do you think this intervention is in line with the priorities of leadership within X DEPARTMENT, including senior leaders and mid-level managers?**
- **To what extent do you feel this intervention will help you to meet expectations from management or leadership about the care you provide in X SERVICE LINE?**
- **What, if any, are the anticipated benefits of this program for the families you serve in X SERVICE LINE?**
