# Supplementary material for: Engaging Parents and Health Care Stakeholders to Inform Development of a Behavioral Intervention Technology to Promote Pediatric Behavioral Health: Mixed Methods Study
Source: JMIR Pediatr Parent. 2021 Oct 5;4(4):e27551. doi: 10.2196/27551 (PMC8527378; doi:10.2196/27551)
Supplement: Multimedia Appendix 2 [file pediatrics_v4i4e27551_app2.pdf]

Multimedia Appendix 2

Parent Quantitative Survey

Start of Block: Screeners

Intro) Thanks for choosing to share your experiences as a parent about important developmental and behavioral topics. Your answers will help us develop a website that shares information and strategies around these issues.

Providing feedback here will not automatically give you access to the materials once they are developed. At the end of the survey, please let us know if you would like to be considered for a user test group once the materials are developed.

Your responses will be kept entirely confidential. Please click the orange arrow below to begin the survey.

Page Break

Q1) Are you a parent or guardian of a child 18 years of age or younger?

- ○ Yes (1)
- ○ No (2)

Skip To: End of Survey If Q1 = No

Page Break

Q2) How many children 18 years of age or younger do you have?

- 1 (1)
- 2 (2)
- 3 (3)
- 4 (4)
- More than 4 (5)

Page Break

Q3) Please indicate the age(s) of your children.

- ☐ 0-5 (1)
- ☐ 6-12 (2)
- ☐ 13-18 (3)

Page Break

Q4) Please rate your level of interest in using online resources to research issues and concerns you may have about children and parenting.

- ○ Not at all interested (1)
- ○ Somewhat interested (2)
- ○ Very interested (3)

Skip To: End of Survey If Q4 = Not at all interested

Page Break

Q5) Which of the following parenting topics would you be most interested in receiving information about? Select up to three.

- ☐ Anxiety in Children (1)
- ☐ Nutrition/Eating (2)
- ☐ Child Safety (3)
- ☐ Behavioral Challenges (4)
- ☐ Speech/Language Skills (5)
- ☐ Mood or Depression in Children (6)
- ☐ Academic Skills and/or Intelligence (7)
- ☐ The Internet and Social Media (8)
- ☐ Parenting Stress (9)
- ☐ Family Communication (10)
- ☐ Social Skills (11)
- ☐ Drugs and Substance Abuse (12)
- ☐ Sex and Sexual Development (13)
- ☐ Motor Skills (14)
- ☐ Independence and Activities of Daily Living (15)

☐ Sleep/Bedtime Routine (16)

☐ Toileting (17)

Page Break

Start of Block: Anxiety in Children

Anxiety_Q1) You indicated that "Anxiety in Children" is a topic you would be interested in. Which of the following statements is true?

○ "Anxiety in Children" has been or is currently a challenging topic for me as a parent. (1)

○ "Anxiety in Children" is not a current challenge, but I would like more information. (2)

Page Break

Anxiety_Q2) Please indicate your interest in learning about each of the following topics pertaining to anxiety in children.

	Yes (1)	No (2)
Frequent worries (1)	○	○
Difficulty separating from parents/caregivers (2)	○	○
Complaining about physical symptoms (e.g., upset stomach, headaches, etc.) (3)	○	○
Fears of very specific things (e.g., spiders, snakes, heights, etc.) (4)	○	○
Avoiding going to school (5)	○	○
Panic attacks (6)	○	○
Repetitive thoughts and/or behaviors (7)	○	○
Worrying about social situations (8)	○	○

Skip To: End of Block If Anxiety_Q2 = Difficulty separating from parents/caregivers

Page Break

Anxiety_Q3) Please select the response category that best represents your personal experience(s) - if any - with each of the following strategies for helping your child(ren) cope with their anxiety.

	Tried and was helpful (1)	Tried but was not helpful (2)	Did not try (3)	N/A (4)
Relaxation strategies (e.g., deep breathing, muscle relaxation, etc.) (1)	○	○	○	○
Expose child to things they fear (2)	○	○	○	○
Encourage child to try new things (3)	○	○	○	○
Comfort child when they're afraid (4)	○	○	○	○
Take child to the doctor for medication (5)	○	○	○	○
Encourage child to stay at home when you're away (6)	○	○	○	○
Give child reassurance that everything is okay (7)	○	○	○	○
Take child for therapy/counseling (8)	○	○	○	○
Limit opportunities for separation (9)	○	○	○	○
Tell child about how emotions are connected to their bodies (10)	○	○	○	○
Encourage child to think differently (11)	○	○	○	○
Encourage child to stop repetitive habits or rituals (12)	○	○	○	○

End of Block: Anxiety in Children

Start of Block: Nutrition/Eating

Nutrition_Q1) You indicated that "Nutrition/Eating" is a topic you would be interested in. Which of the following statements is true?

- "Nutrition/Eating" has been or is currently a challenging topic for me as a parent. (1)
- "Nutrition/Eating" is not a current challenge, but I would like more information. (2)

Page Break

Nutrition_Q2) Please indicate your interest in learning about each of the following topics pertaining to nutrition/eating.

	Yes (1)	No (2)
Picky/selective eating (1)	○	○
Eating too much unhealthy food (2)	○	○
Unhealthy weight management (e.g., food restriction, throwing up after eating, excessive exercise, etc.) (3)	○	○
Maintaining a healthy weight (4)	○	○
Refusing to eat (e.g., throwing a tantrum) (5)	○	○

Skip To: End of Block If Nutrition_Q2 = Eating too much unhealthy food

Nutrition_Q3 Please select the response that best represents your personal experience(s) - if any - with each of the following strategies for helping your child(ren) with their nutrition/eating.

	Tried and was helpful (1)	Tried but was not helpful (2)	Did not try (3)	N/A (4)
Punish child for not eating (1)	○	○	○	○
Allow child to eat what they want after taking at least one bite of healthy food (2)	○	○	○	○
Praise child for eating (3)	○	○	○	○
Give a child a reward for eating (e.g., extra TV time, staying up late, etc.) (4)	○	○	○	○
Let child choose food options (5)	○	○	○	○
Make separate meals for my child (6)	○	○	○	○
Do not keep unhealthy foods in the house (7)	○	○	○	○
Portion control of unhealthy foods (8)	○	○	○	○
Intensive interventions (e.g., bariatric surgery, low-calorie diet, residential camp, etc.) (9)	○	○	○	○
Strictly monitor food intake/output (10)	○	○	○	○

Ignore inappropriate mealtime behaviors (e.g., tantrums) (11)	○	○	○	○
Talk to health care provider about medical concerns (12)	○	○	○	○
Allow my child to eat whatever they want (13)	○	○	○	○
Give medication to my child for weight gain/loss (14)	○	○	○	○
Provide nutrition supplements (e.g., PediaSure) (15)	○	○	○	○
Keep a set meal/snack schedule (16)	○	○	○	○
Offer new foods frequently, even if my child hasn't liked them before (17)	○	○	○	○
Limit high calorie drinks (e.g., juice, soda, etc.) (18)	○	○	○	○

End of Block: Nutrition/Eating

Start of Block: Behavior Challenges

Behavior_Q1) You indicated that "Behavioral Challenges" is a topic you would be interested in. Which of the following statements is true?

○ "Behavioral Challenges" has been or is currently a challenging topic for me as a parent. (1)

○ "Behavioral Challenges" is not a current challenge, but I would like more information. (2)

Page Break

Behavior_Q2) Please indicate your interest in learning about each of the following topics pertaining to behavioral challenges.

	Yes (1)	No (2)
Not following instructions (1)	○	○
Breaking house rules (2)	○	○
Aggression toward others (e.g., hitting, kicking, throwing things, etc.) (3)	○	○
Frequent tantrums, meltdowns, or angry outbursts (4)	○	○
Not completing responsibilities (e.g., homework, chores, daily routine, etc.) (5)	○	○

Skip To: End of Block If Behavior_Q2 = Breaking house rules

Page Break

Behavior_Q3) Please select the response category that best represents your personal experience(s) - if any - with each of the following strategies for helping your child(ren) with behavioral challenges.

	Tried and was helpful (1)	Tried and was not helpful (2)	Did not try (3)	N/A (4)
Repeat directions (1)	○	○	○	○
Give child time-out/send to their room (2)	○	○	○	○
Count to three or give a series of warnings (3)	○	○	○	○
Spanking or other form of physical punishment (4)	○	○	○	○
Offer child a reward or incentive if expectations are met (5)	○	○	○	○
Raise my voice (6)	○	○	○	○
Ignore child's misbehavior (7)	○	○	○	○
Ground child from privileges (e.g., no going out, no electronics, etc.) (8)	○	○	○	○
Threaten child with punishments if behavior does not improve (9)	○	○	○	○
Use a regular reward program with daily/weekly rewards (10)	○	○	○	○
Physically restrain my child (11)	○	○	○	○

Talk about why it is important to meet expectations (12)	○	○	○	○
Help child to complete responsibilities (13)	○	○	○	○

End of Block: Behavior Challenges

Start of Block: Speech/Language Skills

Speech_Q1) You indicated that "Speech/Language Skills" is a topic you would be interested in. Which of the following statements is true?

○ "Speech/Language/Skills" has been or is currently a challenging topic for me as a parent. (1)

○ "Speech/Language Skills" is not a current challenge, but I would like more information. (2)

Page Break

Speech_Q2) Please indicate your interest in learning about each of the following topics pertaining to speech/language.

	Yes (1)	No (2)
Pronouncing words (e.g., saying words clearly) (1)	○	○
Understanding what others say (2)	○	○
Frequent grammatical errors (3)	○	○
Mixing up words (4)	○	○
Difficulty learning new words (5)	○	○
Stuttering/false starts (6)	○	○

Skip To: End of Block If Speech_Q2 = Understanding what others say

Page Break

Speech_Q3) Please select the response category that best represents your personal experience(s) - if any - with each of the following strategies for helping your child(ren) with their speech/language skills.

	Tried and was helpful (1)	Tried but was not helpful (2)	Did not try (3)	N/A (4)
Talk for my child so that others can understand them (1)	○	○	○	○
Receive services from a speech/language therapist (2)	○	○	○	○
Correct speech and have my child repeat it (3)	○	○	○	○
Praise child when speech improves (4)	○	○	○	○
Talk to a health care provider about my concerns (5)	○	○	○	○
Use visuals to communicate (6)	○	○	○	○
Use a pointing system to communicate (7)	○	○	○	○
Use visual pictures of a task to complete (8)	○	○	○	○
Practice vocabulary with my child (9)	○	○	○	○
Talk with my child to provide them with more vocabulary (10)	○	○	○	○
Praise my child's vocabulary (11)	○	○	○	○
Read to my child regularly using books with new vocabulary (12)	○	○	○	○

Tell my child to stop/slow down when stuttering (13)	○	○	○	○
Play turn-taking games (e.g., peek-a-boo) (14)	○	○	○	○
Teach my child sign language (15)	○	○	○	○

End of Block: Speech/Language Skills

Start of Block: Mood or Depression in Children

Mood_Q1) You indicated that "Mood or Depression in Children" is a topic you would be interested in. Which of the following statements is true?

○ "Mood or Depression in Children" has been or is currently a challenging topic for me as a parent. (1)

○ "Mood or Depression in Children" is not a current challenge, but I would like more information. (2)

Page Break

Mood_Q2) Please indicate your interest in learning about each of the following topics pertaining to mood or depression in children.

	Yes (1)	No (2)
Irritability/anger (1)	○	○
Loss of interest in activities child used to enjoy (2)	○	○
Seeming down or lonely (3)	○	○
Sleeping too much/too little (4)	○	○
Low self-esteem/negative self-talk (5)	○	○

Skip To: End of Block If Mood_Q2 = Loss of interest in activities child used to enjoy

Page Break

Mood_Q3) Please select the response category that best represents your personal experience(s) - if any - with each of the following strategies for helping your child(ren) cope with their mood or depression.

	Tried and was helpful (1)	Tried but was not helpful (2)	Did not try (3)	N/A (4)
Tell child to stop doing things that make them irritated (1)	○	○	○	○
Punish child (2)	○	○	○	○
Talk to child about why they're upset (3)	○	○	○	○
Take child to a mental health professional (4)	○	○	○	○
Take child to a doctor for assessment and/or medication (5)	○	○	○	○
Encourage child to engage in fun activities (6)	○	○	○	○
Teach child how to relax before bedtime (7)	○	○	○	○
Tell child to go to bed earlier (8)	○	○	○	○
Allow child to take naps or sleep in on weekends (9)	○	○	○	○
Give child reasons to be happy (10)	○	○	○	○
Give child examples of how they're supported (11)	○	○	○	○
Tell child to stop judging themselves harshly (12)	○	○	○	○

Encourage child to speak positively about their strengths (13)	○	○	○	○
Identify a mentor for my child to talk to (e.g., coach, teacher, family member, etc.) (14)	○	○	○	○

End of Block: Mood or Depression in Children

Start of Block: Academic Skills and/or Intelligence

Academic_Q1) You indicated that "Academic Skills and/or Intelligence" is a topic you would be interested in. Which of the following statements is true?

○ "Academic Skills and/or Intelligence" has been or is currently a challenging topic for me as a parent. (1)

○ "Academic Skills and/or Intelligence" is not a current challenge, but I would like more information. (2)

Page Break

Academic_Q2) Please indicate your interest in learning about each of the following topics pertaining to academic skills and/or intelligence.

	Yes (1)	No (2)
Focus (1)	o	o
Finishing tasks (2)	o	o
Organization (3)	o	o
Planning (4)	o	o
Learning new skills (e.g., ABCs, counting, reading, etc.) (5)	o	o
Understanding numbers and/or math (6)	o	o
Learning the alphabet and/or reading (7)	o	o

Skip To: End of Block If Academic_Q2 = Finishing tasks

Page Break

Academic_Q3) Please select the response category that best represents your personal experience(s) - if any - with each of the following strategies for helping your child(ren) with their academic skills and/or intelligence.

	Tried and was helpful (1)	Tried but was not helpful (2)	Did not try (3)	N/A (4)
Limit distractions (e.g., turn off TV, take away toys, etc.) (1)	○	○	○	○
Verbally praise child (2)	○	○	○	○
Give reward to child (3)	○	○	○	○
Talk to a health care provider about the concerns (4)	○	○	○	○
Talk to my child's teacher about the concerns (5)	○	○	○	○
Receive early intervention/Special Education services (6)	○	○	○	○
Punish child when things are not done right (7)	○	○	○	○
Rehearse information with my child to help them remember (8)	○	○	○	○
Practice math or reading with my child at home (9)	○	○	○	○
Punishment for not understanding numbers and/or math (10)	○	○	○	○
Let my child learn on their own (11)	○	○	○	○
Create a visual schedule/reminder for my child to complete personal care tasks (12)	○	○	○	○

Complete self-help tasks for my child (13)	○	○	○	○

End of Block: Academic Skills and/or Intelligence

Start of Block: The Internet and social media

Internet_Q1) You indicated that "the Internet and Social Media" is a topic you would be interested in. Which of the following statements is true?

○ "The Internet and Social Media" has been or is currently a challenging topic for me as a parent. (1)

○ "The Internet and Social Media" is not a current challenge, but I would like more information. (2)

Page Break

Internet_Q2 Please indicate your interest in learning about each of the following topics pertaining to the Internet and social media.

	Yes (1)	No (2)
Setting limits around social media use (1)	○	○
Cyber bullying (2)	○	○
Online safety (3)	○	○
Sending and/or receiving inappropriate content online (4)	○	○
Protecting the privacy of personal information on social media (5)	○	○

Skip To: End of Block If Internet_Q2 = Cyber bullying

Page Break

Internet_Q3) Please select the response category that best represents your personal experience(s) - if any - with each of the following strategies for monitoring your child(ren)'s Internet and/or social media use.

	Tried and was helpful (1)	Tried but was not helpful (2)	Did not try (3)	N/A (4)
Punishment for inappropriate online behavior (1)	○	○	○	○
Take away devices used for access (e.g., tablet, laptop, smart phone, etc.) (2)	○	○	○	○
Set daily screen time limits (3)	○	○	○	○
Increased monitoring of social media use (4)	○	○	○	○
Encourage child to spend time doing non-social media activities (5)	○	○	○	○
Contact the cyber bully or family of the cyber bully (6)	○	○	○	○
Contact my child's school to address the cyber bullying (7)	○	○	○	○
Teach child how to communicate safely online (8)	○	○	○	○
Prepare child for how to respond appropriately to cyber bullies/other online predators (9)	○	○	○	○

Contact police/local authorities about the cyber bullying/online harassment (10)	○	○	○	○

End of Block: The Internet and social media

Start of Block: Parenting Stress

ParentStress_Q1) You indicated that "Parenting Stress" is a topic you would be interested in. Which of the following statements is true?

○ "Parenting Stress" has been or is currently a challenging topic for me as a parent. (1)

○ "Parenting Stress" is not a current challenge, but I would like more information. (2)

Page Break

ParentStress_Q2) Please indicate your interest in learning about each of the following topics pertaining to parenting stress.

	Yes (1)	No (2)
Coping with difficult emotions or thoughts (1)	○	○
Balancing self-care with other responsibilities (2)	○	○
Dealing with self-criticism (3)	○	○
Dealing with criticism from others/society (4)	○	○
Getting support from the community (5)	○	○
Getting support from other caregivers (6)	○	○

Skip To: End of Block If ParentStress_Q2 = Balancing self-care with other responsibilities

Page Break

ParentStress_Q3) Please select the response category that best represents your personal experience(s) - if any - with each of the following strategies for helping you cope with parenting stress.

	Tried and was helpful (1)	Tried but was not helpful (2)	Did not try (3)	N/A (4)
Reach out to loved ones (1)	○	○	○	○
Increase engagement in activities I enjoy (2)	○	○	○	○
Tell myself that these feelings are normal (3)	○	○	○	○
Tell myself that these feelings will go away (4)	○	○	○	○
Opt out of situations/activities where difficult thoughts/emotions are likely to occur (5)	○	○	○	○
Distract myself (e.g., TV, Internet, phone, etc.) (6)	○	○	○	○
Use strategies to alleviate the thoughts/emotions (e.g., overeating, oversleeping, substance use, etc.) (7)	○	○	○	○
Try to keep difficult thoughts/emotions out of my mind (8)	○	○	○	○
Take medication to improve my mood (9)	○	○	○	○
Take time away from responsibilities to de-stress (10)	○	○	○	○
Participate in therapy/counseling (11)	○	○	○	○

End of Block: Parenting Stress

Start of Block: Family Communication

FamilyCx_Q1) You indicated that "Family Communication" is a topic you would be interested in. Which of the following statements is true?

○ "Family Communication" has been or is currently a challenging topic for me as a parent. (1)

○ "Family Communication" is not a current challenge, but I would like more information. (2)

Page Break

FamilyCx_Q2 Please indicate your interest in learning about each of the following topics pertaining to family communication.

	Yes (1)	No (2)
Frequent arguments/conflict (1)	○	○
Lack of open communication (2)	○	○
Toxic communication (e.g., yelling, saying hateful things, threatening, etc. (3)	○	○
Protecting children from parental conflict (4)	○	○
Finding common ground with other caregivers on parenting strategies (5)	○	○
Effective strategies for co-parenting (6)	○	○

Skip To: End of Block If FamilyCx_Q2 = Lack of open communication

FamilyCx_Q3) Please select the response category that best represents your personal experience(s) - if any - with each of the following strategies for helping your child(ren) with family communication.

	Tried and was helpful (1)	Tried but was not helpful (2)	Did not try (3)	N/A (4)
Avoid correcting my child to avoid arguments (1)	○	○	○	○
Avoid criticizing my child to avoid arguments (2)	○	○	○	○
Count to 3 or give a series of warnings (3)	○	○	○	○
Give my child a time-out or equivalent (e.g., grounding child from privileges) (4)	○	○	○	○
Describe to my child how I feel about the situation (5)	○	○	○	○
Spank my child's bottom (6)	○	○	○	○
Consult a professional (e.g., doctor, therapist, etc.) (7)	○	○	○	○
Raise my voice/yell at my child (8)	○	○	○	○
Talk calmly with my child about the situation (9)	○	○	○	○
Use ultimatums with my child (10)	○	○	○	○
Threaten my child with punishments (11)	○	○	○	○

Talk regularly with my child about positive things (12)	○	○	○	○
Tell my child why they have to do things (13)	○	○	○	○
Talk to my child about what we value as parents (14)	○	○	○	○
Talk to my child about what we as parents want for their future (15)	○	○	○	○

End of Block: Family Communication

Start of Block: Social Skills

SocialSkills_Q1) You indicated that "Social Skills" is a topic you would be interested in. Which of the following statements is true?

○ "Social Skills" has been or is currently a challenging topic for me as a parent. (1)

○ "Social Skills" is not a current challenge, but I would like more information. (2)

Page Break

SocialSkills_Q2) Please indicate your interest in learning about each of the following topics pertaining to social skills.

	Yes (1)	No (2)
Communicating with others (1)	o	o
Sharing/taking turns (2)	o	o
Understanding how others feel (i.e., empathy) (3)	o	o
Playing with other children (4)	o	o
Aggression during social interactions (5)	o	o

Skip To: End of Block If SocialSkills_Q2 = Sharing/taking turns

Page Break

SocialSkills_Q3) Please select the response category that best represents your personal experience(s) - if any - with each of the following strategies for helping your child(ren) with their social skills.

	Tried and was helpful (1)	Tried but was not helpful (2)	Did not try (3)	N/A (4)
Talk for my child when introducing them to others (1)	○	○	○	○
Talk to a therapist or other health care professional about my child's social skills (2)	○	○	○	○
Verbally praise my child for good social skills (3)	○	○	○	○
Give my child a reward for good social skills (4)	○	○	○	○
Punish my child for aggression in social situations (e.g., hitting or kicking others) (5)	○	○	○	○
Punish my child for poor social skills (6)	○	○	○	○
Model/show my child what to do in social situations (7)	○	○	○	○
Take my child to public places to interact with others (e.g., park, library, etc.) (8)	○	○	○	○
Play turn-taking games with my child to practice social skills (9)	○	○	○	○

Talk to my child's teacher about my concerns (10)	○	○	○	○
Describe emotions in other people to my child (11)	○	○	○	○
Remove distractions so my child can focus on playing with others (e.g., electronics) (12)	○	○	○	○

End of Block: Social Skills

Start of Block: Drugs and Substance Abuse

Drugs_Q1) You indicated that "Drugs and Substance Abuse" is a topic you would be interested in. Which of the following statements is true?

○ "Drugs and Substance Abuse" has been or is currently a challenging topic for me as a parent. (1)

○ "Drugs and Substance Abuse" is not a current challenge, but I would like more information. (2)

Page Break

Drugs_Q2) Please indicate your interest in learning about each of the following topics pertaining to drugs and substance abuse.

	Yes (1)	No (2)
Tobacco products (e.g., nicotine, electronic cigarettes, vaping, etc.) (1)	○	○
Marijuana (e.g., THC, edibles, CBD products, etc.) (2)	○	○
Alcohol (3)	○	○
Opiates (e.g., heroin, Oxycontin, etc.) (4)	○	○
Amphetamines (e.g., cocaine, crystal meth, stimulant medications such as Adderall that are not prescribed by a doctor, etc.) (5)	○	○

Skip To: End of Block If Drugs_Q2 = Marijuana (e.g., THC, edibles, CBD products, etc.)

Page Break

Drugs_Q3) Please select the response category that best represents your personal experience(s) - if any - with each of the following strategies for helping your child(ren) with drugs and substance abuse.

	Tried and was helpful (1)	Tried but was not helpful (2)	Did not try (3)	N/A (4)
Talk to my child about negative consequences of using the substance (1)	○	○	○	○
Take the substance away from my child (2)	○	○	○	○
Punish my child for using the substance (3)	○	○	○	○
Take my child to a mental health professional (4)	○	○	○	○
Allow use of substances under supervision (5)	○	○	○	○
Give child a urine or blood test to check for substances (6)	○	○	○	○

End of Block: Drugs and Substance Abuse

Start of Block: Sex and Sexual Development

Sex_Q1 You indicated that "Sex and Sexual Development" is a topic you would be interested in. Which of the following statements is true?

○ "Sex and Sexual Development" has been or is currently a challenging topic for me as a parent. (1)

○ "Sex and Sexual Development" is not a current challenge, but I would like more information. (2)

Sex_Q2) Please indicate your level of interest in learning about each of the following topics pertaining to sex and sexual development.

	Yes (1)	No (2)
Understanding bodily changes that come with puberty (1)	○	○
Gender identity (2)	○	○
Sexual orientation (3)	○	○
Teaching healthy sexual development (4)	○	○
Teaching social norms around sex (5)	○	○
Teaching privacy regarding sex (6)	○	○
Safe sex (7)	○	○
Family planning (8)	○	○
Healthy romantic relationships (9)	○	○

Skip To: End of Block If Sex_Q2 = Gender identity

Page Break

Sex_Q3) Please select the response category that best represents your personal experience(s) - if any - with each of the following strategies for helping your child(ren) cope with their sexual development and/or learn about sex.

	Tried and was helpful (1)	Tried but was not helpful (2)	Did not try (3)	N/A (4)
Talk to my child about puberty (1)	○	○	○	○
Talk to my child about sex (2)	○	○	○	○
Encourage child to ask questions about puberty (3)	○	○	○	○
Encourage child to ask questions about sex (4)	○	○	○	○
Talk to my health care provider (5)	○	○	○	○
Provide an open space for my child to talk to family members without fear of judgment/punishment (6)	○	○	○	○
Have my child talk to community leaders for guidance (7)	○	○	○	○
Punish my child for inappropriate behavior (8)	○	○	○	○
Talk to my child's teacher (9)	○	○	○	○
Talk to my child about who may see their "private areas" (e.g., medical provider) (10)	○	○	○	○
Ignore my child when they show their "private areas" to others inappropriately (11)	○	○	○	○
Punish my child for having sex (12)	○	○	○	○

Punish my child for not practicing safe sex (13)	○	○	○	○
Encourage safe sex practices (14)	○	○	○	○
Provide my child with condoms/other birth control methods (15)	○	○	○	○
Talk to my child about when/where it's appropriate to masturbate (16)	○	○	○	○
Provide my child with health literature about masturbating in private (17)	○	○	○	○
Talk to my child about healthy boundaries with a romantic partner (18)	○	○	○	○
Talk to my child about how to deal with romantic partner violence (19)	○	○	○	○
Restrict my child's ability to date (20)	○	○	○	○
Discuss risky sexual behaviors with my child (21)	○	○	○	○

End of Block: Sex and Sexual Development

Start of Block: Motor Skills

MotorSkills_Q1) You indicated that "Motor Skills" is a topic you would be interested in. Which of the following statements is true?

○ "Motor Skills" has been or is currently a challenging topic for me as a parent. (1)

○ "Motor Skills" is not a current challenge, but I would like more information. (2)

Page Break

MotorSkills_Q2) Please indicate your interest in learning about each of the following topics pertaining to motor skills.

	Yes (1)	No (2)
Walking on their own (1)	○	○
Handwriting (2)	○	○
Walking on their toes (3)	○	○
Fine motor skills (e.g., buttons, zippers, picking up objects, etc.) (4)	○	○
Dominant hand (e.g., still using both hands, not clearly right- or left-handed, etc.) (5)	○	○

Skip To: End of Block If MotorSkills_Q2 = Handwriting

Page Break

MotorSkills_Q3) Please select the response category that best represents your personal experience(s) - if any - with each of the following strategies for helping your child(ren) with their motor skills.

	Tried and was helpful (1)	Tried but was not helpful (2)	Did not try (3)	N/A (4)
Limit distractions to help child pay attention (e.g., turn off TV, take away toys, etc.) (1)				
Show excitement/praise child when they do something right (2)				
Talk to my child's health care provider about the concerns (3)				
Talk to my child's teacher about the concerns (4)				
Use standing toys or walkers to strengthen walking (5)				
Receive occupational therapy (6)				
Give child a time-out when something is not done right (7)				
Take away child's toys when something is not done right (8)				
Put my hand over my child's hand while they write (9)				
Demonstrate how to do a skill (10)				

Practice skills with my child (11)	o	o	o	o
Play games with my child to help them walk on the whole foot (12)	o	o	o	o
Do things requiring fine motor skills for my child (e.g., help them write, dress, button, etc.) (13)	o	o	o	o
Play games with my child to help them practice fine motor skills (14)	o	o	o	o

End of Block: Motor Skills

Start of Block: Independence and Activities of Daily Living

Independence_Q1) You indicated that "Independence and Activities of Daily Living" is a topic you would be interested in. Which of the following statements is true?

o "Independence and Activities of Daily Living" has been or is currently a challenging topic for me as a parent. (1)

o "Independence and Activities of Daily Living" is not a current challenge, but I would like more information. (2)

Page Break

Independence_Q2) Please indicate your interest in learning about each of the following topics pertaining to independence and activities of daily living.

	Yes (1)	No (2)
Requesting or describing needs (e.g., asking for snacks, to go to the bathroom/have diaper changed, etc.) (1)	○	○
Independence with self-help tasks (e.g., dressing, bathing, going to the bathroom, etc.) (2)	○	○
What to do in an emergency (e.g., get help, call 911, etc.) (3)	○	○
Solving everyday problems independently (e.g., when something doesn't go right, when they run out of something, etc.) (4)	○	○
Understanding money (e.g., needing money to buy things, how to save money, etc.) (5)	○	○

Skip To: End of Block If Independence_Q2 = Independence with self-help tasks (e.g., dressing, bathing, going to the bathroom, etc.)

Page Break

Independence_Q3) Please select the response category that best represents your personal experience(s) - if any - with each of the following strategies for helping your child(ren) with independence and the activities of daily living.

	Tried and was helpful (1)	Tried but was not helpful (2)	Did not try (3)	N/A (4)
Coach/talk to my child about learning how to be independent (1)	○	○	○	○
Show excitement/praise my child when they complete tasks independently (2)	○	○	○	○
Show my child how to do things (3)	○	○	○	○
Practice how to do things repetitively with my child (4)	○	○	○	○
Consult with my pediatrician/other medical provider (5)	○	○	○	○
Make a home emergency safety plan that's posted somewhere my child can see it (e.g., refrigerator) (6)	○	○	○	○
Develop a reward system/behavior chart to motivate my child to complete tasks independently (7)	○	○	○	○

Teach my child how to manage finances (e.g., balancing a checkbook, using a credit/debit card appropriately, etc.) (8)	○	○	○	○
Have my child help me with household tasks (e.g., grocery shopping) (9)	○	○	○	○
Let my child complete tasks at their own pace (10)	○	○	○	○
Complete self-help tasks for my child (e.g., dressing, bathing, etc.) (11)	○	○	○	○

End of Block: Independence and Activities of Daily Living

Start of Block: Sleep/Bedtime Routine

Sleep_Q1) You indicated that "Sleep/Bedtime Routine" is a topic you would be interested in. Which of the following statements is true?

○ "Sleep/Bedtime Routine" has been or is currently a challenging topic for me as a parent. (1)

○ "Sleep/Bedtime Routine" is not a current challenge, but I would like more information. (2)

Page Break

Sleep_Q2) Please indicate your interest in learning about each of the following topics pertaining to sleep/bedtime routine.

	Yes (1)	No (2)
Bedtime routine (1)	○	○
Falling asleep independently at bedtime (2)	○	○
Sleeping through the night (3)	○	○
Common sleep problems/resolutions (4)	○	○
Relationship between sleep and health (5)	○	○

Skip To: End of Block If Sleep_Q2 = Falling asleep independently at bedtime

Page Break

Sleep_Q3) Please select the response category that best represents your personal experience(s) - if any - with each of the following strategies for helping your child(ren) with their sleep/bedtime routine.

	Tried and was helpful (1)	Tried but was not helpful (2)	Did not try (3)	N/A (4)
Stay in my child's room until they fall asleep (1)	o	o	o	o
Allow my child to sleep in my bed if they don't fall asleep in theirs (2)	o	o	o	o
Sit on my child's bed until they fall asleep (3)	o	o	o	o
Give my child an over-the-counter sleep aid (e.g., melatonin, Benadryl, etc.) (4)	o	o	o	o
Allow my child to "cry it out" until they fall asleep on their own (5)	o	o	o	o
Offer my child a reward for staying in bed at bedtime/throughout the night (6)	o	o	o	o
Complete an evaluation with a sleep specialist (7)	o	o	o	o
Discuss my child's sleep concerns with a sleep specialist (8)	o	o	o	o
Immediately take my child back to their room if they get up (9)	o	o	o	o
Allow my child to watch TV or another electronic device until they fall asleep (10)	o	o	o	o

End of Block: Sleep/Bedtime Routine

Start of Block: Toileting

Toileting_Q1) You indicated that "Toileting" is a topic you would be interested in. Which of the following statements is true?

○ "Toileting" has been or is currently a challenging topic for me as a parent. (1)

○ "Toileting" is not a current challenge, but I would like more information. (2)

Page Break

Toileting_Q2) Please indicate your interest in learning about each of the following topics pertaining to toileting.

	Yes (1)	No (2)
Toilet training (1)	○	○
Bedwetting (2)	○	○
Daytime bowel accidents (3)	○	○
Daytime bladder accidents (4)	○	○
Health problems that affect toileting (e.g., constipation, diarrhea, reflux, etc.) (5)	○	○

Skip To: End of Survey If Toileting_Q2 = Bedwetting

Page Break

Toileting_Q3) Please select the response category that best represents your personal experience(s) - if any - with each of the following strategies for helping your child(ren) with toileting.

	Tried and was helpful (1)	Tried but was not helpful (2)	Did not try (3)	N/A (4)
Read books with my child about using the toilet (1)	○	○	○	○
Watch videos with my child about using the toilet (2)	○	○	○	○
Have my child sit on the toilet for 5-10 minute intervals (e.g., every 2-3 hours) (3)	○	○	○	○
Have my child urinate twice during the same bathroom visit (4)	○	○	○	○
Verbally praise my child when they pee/poop in the toilet (5)	○	○	○	○
Give my child a reward when they pee/poop in the toilet (6)	○	○	○	○
Do activities with my child while they're on the toilet (e.g., read stories, play games, etc.) (7)	○	○	○	○
Keep my child naked for periods of time (i.e., the bare bottom method) (8)	○	○	○	○

Permit my child to have only very little or no fluids after dinner to reduce the likelihood of nighttime accidents (9)	○	○	○	○
Try to ensure my child avoids caffeine (10)	○	○	○	○
Give my child foods with fiber (11)	○	○	○	○
Wake my child up to go to the bathroom at night (12)	○	○	○	○
Reward my child for staying dry at night (13)	○	○	○	○
Use bedwetting alarms (14)	○	○	○	○
Have my child drink lots of fluid during the day so they can practice peeing (15)	○	○	○	○
Give medication to my child (16)	○	○	○	○
Give laxatives to my child (17)	○	○	○	○
Talk to a health care provider about my concerns (18)	○	○	○	○

End of Block: Toileting

Start of Block: Final Question

FinalQ_1) Please let us know of any additional topics you'd like to see in a guided self-help resource for parents.

____

FinalQ_2 Would you like to participate in a user test group once the materials have been developed? If yes, you will receive a follow-up email when the time comes.

- ○ Yes (1)
- ○ No (3)

End of Block: Final Question
