# Supplementary material for: Engaging Parents and Health Care Stakeholders to Inform Development of a Behavioral Intervention Technology to Promote Pediatric Behavioral Health: Mixed Methods Study
Source: JMIR Pediatr Parent. 2021 Oct 5;4(4):e27551. doi: 10.2196/27551 (PMC8527378; doi:10.2196/27551)
Supplement: Multimedia Appendix 3 [file pediatrics_v4i4e27551_app3.pdf]

Multimedia Appendix 3

Table S1. Anxiety in Children

Anxiety Past/Current or More Information	n	%
"Anxiety in Children" has been or is currently a challenging topic for me as a parent.	79	11%
"Anxiety in Children" is not a current challenge, but I would like more information.	32	29%

Anxiety Concerns	n	%
Frequent worries	86	84%
Worrying about social situations	73	72%
Complaining about physical symptoms (e.g., upset stomach, headaches, etc.)	68	67%
Repetitive thoughts and/or behaviors	47	46%
Panic attacks	44	43%
Fears of very specific things (e.g., spiders, snakes, heights, etc.)	42	41%
Difficulty separating from parents/caregivers	40	39%
Avoiding going to school	33	32%

Anxiety Strategies	Tried and was helpful		Tried but was not helpful		Did not try		N/A	
	n	%	n	%	n	%	n	%
Comfort child when they're afraid	63	71%	17	19%	0	0%	9	10%
Give child reassurance that everything is okay	55	62%	29	33%	1	1%	4	4%
Encourage child to try new things	49	55%	26	29%	3	3%	11	12%
Relaxation strategies (e.g., deep breathing, muscle relaxation, etc.)	41	46%	21	24%	19	21%	8	9%
Encourage child to think differently	39	44%	35	39%	5	6%	10	11%
Take child for therapy/counseling	38	43%	10	11%	28	31%	13	15%
Tell child about how emotions are connected to their bodies	31	35%	17	19%	30	34%	11	12%
Expose child to things they fear	17	19%	19	21%	29	32%	25	28%
Take child to the doctor for medication	16	18%	8	9%	48	54%	17	19%
Encourage child to stay at home when you're away	15	17%	12	13%	21	24%	41	46%
[a]Limit opportunities for separation	14	16%	11	12%	18	20%	46	52%
[a]Encourage child to stop repetitive habits or rituals	11	12%	28	31%	9	10%	41	46%

[a]Inappropriate strategy

Multimedia Appendix 3

Table S2. Behavioral Challenges

**Behavior Challenges Past/Current or More Information**	n	%						
"Behavioral Challenges" has been or is currently a challenging topic for me as a parent.	71	75%						
"Behavioral Challenges" is not a current challenge, but I would like more information.	24	25%						
**Behavioral Challenges Concerns**	n	%						
Not following instructions	72	77%						
Frequent tantrums, meltdowns, or angry outbursts	71	76%						
Not completing responsibilities (e.g., homework, chores, daily routine, etc.)	67	71%						
Breaking house rules	57	61%						
Aggression toward others (e.g., hitting, kicking, throwing things, etc.)	53	56%						
**Behavioral Challenges Stratagies**	Tried and was helpful		Tried but was not helpful		Did not try		N/A	
	#	%	#	%	#	%	#	%
Help child to complete responsibilities	55	64%	24	28%	6	7%	1	1%
Offer child a reward or incentive if expectations are met	53	62%	25	29%	5	6%	3	3%
[a]Repeat directions	44	51%	37	43%	2	2%	3	3%
Ground child from privileges (e.g., no going out, no electronics, etc.)	40	47%	29	34%	8	9%	9	10%
Give child time-out/send to their room	38	44%	39	45%	6	7%	3	3%
Count to three or give a series of warnings	37	43%	36	42%	12	14%	1	1%
Talk about why it is important to meet expectations	35	41%	38	44%	8	9%	5	6%
[a]Threaten child with punishments if behavior does not improve	27	31%	43	50%	11	13%	5	6%
[a]Raise my voice	26	30%	57	66%	2	2%	1	1%
Use a regular reward program with daily/weekly rewards	26	30%	29	34%	27	31%	4	5%
Ignore child's misbehavior	20	23%	41	48%	23	27%	2	2%
[a]Physically restrain my child	13	15%	13	15%	48	56%	12	14%
[a]Spanking or other form of physical punishment	10	12%	13	15%	52	60%	11	13%

[a]Inappropriate strategy

Multimedia Appendix 3

Table S3. Nutrition/Eating

**Nutrition/Eating Past/Current or More Information**	n	%
"Nutrition/Eating" has been or is currently a challenging topic for me as a parent.	55	54%
"Nutrition/Eating" is not a current challenge, but I would like more information.	47	46%

**Nutrition/Eating Concerns**	n	%
Picky/selective eating	75	71%
Maintaining a healthy weight	64	61%
Eating too much unhealthy food	63	60%
Refusing to eat (e.g., throwing a tantrum)	33	31%
Unhealthy weight management (e.g., food restriction, throwing up after eating, excessive exercise, etc.)	17	16%

**Nutrition/Eating Strategies**	Tried and was helpful		Tried but was not helpful		Did not try		N/A	
	#	%	#	%	#	%	#	%
Portion control of unhealthy foods	45	53%	17	20%	8	9%	15	18%
Praise child for eating	38	45%	14	16%	8	9%	25	29%
Let child choose food options	36	42%	25	29%	10	12%	15	17%
[a]Make separate meals for my child	29	35%	15	18%	24	29%	16	19%
Do not keep unhealthy foods in the house	21	25%	19	22%	26	31%	19	22%
Talk to health care provider about medical concerns	17	20%	14	17%	24	29%	29	35%
Allow child to eat what they want after taking at least one bite of healthy food	16	19%	11	13%	32	38%	26	31%
Give a child a reward for eating (e.g., extra TV time, staying up late, etc.)	14	16%	10	12%	34	40%	27	32%
Ignore inappropriate mealtime behaviors (e.g., tantrums)	12	14%	17	20%	17	20%	40	47%
Strictly monitor food intake/output	11	13%	10	12%	35	42%	28	33%
[a]Allow my child to eat whatever they want	9	11%	14	17%	36	43%	24	29%
[a]Punish child for not eating	2	2%	5	6%	46	54%	32	38%
Intensive interventions (e.g., bariatric surgery, low-calorie diet, residential camp, etc.)	0	0%	0	0%	37	44%	48	56%

[a]Inappropriate strategy

Multimedia Appendix 3

Table S4. Mood or Depression

**Mood or Depression Past/Current or More Information**	n	%
"Mood or Depression" has been or is currently a challenging topic for me as a parent.	51	62%
"Mood or Depression" is not a current challenge, but I would like more information.	31	38%

**Mood or Depression Concerns**	n	%
Irritability/anger	60	76%
Low self-esteem/negative self-talk	59	75%
Seeming down or lonely	57	72%
Sleeping too much/too little	37	47%
Loss of interest in activities child used to enjoy	29	37%

**Mood or Depression Strategies**	Tried and was helpful		Tried but was not helpful		Did not try		N/A	
	#	%	#	%	#	%	#	%
Talk to child about why they're upset	38	54%	27	38%	0	0%	6	8%
Encourage child to engage in fun activities	36	51%	23	32%	4	6%	8	11%
Give child examples of how they're supported	36	51%	22	31%	6	8%	7	10%
Encourage child to speak positively about their strengths	35	49%	17	24%	12	17%	7	10%
Give child reasons to be happy	34	48%	20	28%	8	11%	9	13%
Allow child to take naps or sleep in on weekends	33	46%	16	23%	6	8%	16	23%
Take child to a mental health professional	31	44%	11	15%	16	23%	13	18%
[a]Tell child to stop judging themselves harshly	28	39%	27	38%	6	8%	10	14%
Take child to a doctor for assessment and/or medication	26	37%	12	17%	15	21%	18	25%
Teach child how to relax before bedtime	21	30%	19	27%	18	25%	13	18%
Tell child to go to bed earlier	21	30%	26	37%	8	11%	16	23%
Tell child to stop doing things that make them irritated	13	18%	29	41%	13	18%	16	23%
[a]Punish child	2	3%	14	20%	30	42%	25	35%

[a]Inappropriate strategy

Multimedia Appendix 3

Ttable S5. The Internet and Social Media

**The Internet and Social Media Past/Current or More Information**	n	%
"The Internet and Social Media" has been or is currently a challenging topic for me as a parent.	51	56%
"The Internet and Social Media" is not a current challenge, but I would like more information.	40	44%

**The Internet and Social Media Concerns**	n	%
Online safety	84	94%
Protecting the privacy of personal information on social media	79	89%
Setting limits around social media use	74	83%
Sending and/or receiving inappropriate content online	62	70%
Cyber bullying	55	62%

**The Internet and Social Media Strategies**	Tried and was helpful		Tried but was not helpful		Did not try		N/A	
	#	%	#	%	#	%	#	%
Encourage child to spend time doing non-social media activities	48	62%	16	21%	0	0%	14	18%
Teach child how to communicate safely online	43	55%	8	10%	9	12%	18	23%
Take away devices used for access (e.g., tablet, laptop, smart phone, etc.)	41	53%	12	15%	9	12%	16	21%
Set daily screen time limits	37	47%	18	23%	12	15%	11	14%
Increased monitoring of social media use	29	37%	14	18%	10	13%	25	32%
Prepare child for how to respond appropriately to cyber bullies/other online predators	23	29%	5	6%	20	26%	30	38%
Punishment for inappropriate online behavior	15	19%	7	9%	6	8%	50	64%
Contact my child's school to address the cyber bullying	6	8%	9	12%	8	10%	55	71%
Contact the cyber bully or family of the cyber bully	3	4%	6	8%	9	12%	59	77%
Contact police/local authorities about the cyber bullying/online harassment	1	1%	4	5%	13	17%	60	77%

Multimedia Appendix 3

Table S6. Parenting Stress

**Parenting Stress Past/Current or More Information**	n	%
"Parenting Stress" has been or is currently a challenging topic for me as a parent.	55	70%
"Parenting Stress" is not a current challenge, but I would like more information.	14	18%
**Parenting Stress Concerns**	n	%
Balancing self-care with other responsibilities	55	83%
Coping with difficult emotions or thoughts	46	70%
Dealing with self-criticism	42	64%
Dealing with criticism from others/society	29	44%
Getting support from the community	26	39%

**Parenting Stress Strategies**	Tried and was helpful		Tried but was not helpful		Did not try		N/A	
	#	%	#	%	#	%	#	%
Increase engagement in activities I enjoy	36	60%	8	13%	11	18%	5	8%
Take time away from responsibilities to de-stress	36	60%	11	18%	10	17%	3	5%
Reach out to loved ones	34	57%	14	23%	10	17%	2	3%
Tell myself that these feelings are normal	31	52%	17	28%	7	12%	5	8%
[a]Distract myself (e.g., TV, Internet, phone, etc.)	30	50%	21	35%	1	2%	8	13%
[a]Opt out of situations/activities where difficult thoughts/emotions are likely to occur	23	38%	14	23%	8	13%	15	25%
[a]Tell myself that these feelings will go away	20	33%	21	35%	12	20%	7	12%
Take medication to improve my mood	20	33%	11	18%	18	30%	11	18%
[a]Try to keep difficult thoughts/emotions out of my mind	17	28%	28	47%	7	12%	8	13%
Participate in therapy/counseling	16	27%	7	12%	28	47%	9	15%
[a]Use strategies to alleviate the thoughts/emotions (e.g., overeating, oversleeping, substance us[illegible]	12	20%	17	28%	19	32%	12	20%

[a]Inappropriate strategy
