# Supplementary material for: Engaging Parents and Health Care Stakeholders to Inform Development of a Behavioral Intervention Technology to Promote Pediatric Behavioral Health: Mixed Methods Study
Source: JMIR Pediatr Parent. 2021 Oct 5;4(4):e27551. doi: 10.2196/27551 (PMC8527378; doi:10.2196/27551)
Supplement: Multimedia Appendix 4 [file pediatrics_v4i4e27551_app4.docx]

Multimedia Appendix 4. Data integration summary.

| Anxiety | Areas of confirmation   - Use of web-based resources: HCS were clear that parents seek web-based resources for behavioral health concerns. Parents indicated that they were interested in web-based resources, had tried various strategies for managing anxiety, and were searching for more anxiety management strategies.   Areas of expansion   - Perceptions/use of resources: HCS noted that unmet parental needs may stem from limited access to resources. Parents endorsed psychotherapy as the most helpful strategy for managing anxiety (80%), but it is unclear how many families have been able to engage in psychotherapy or to what extent psychotherapy has led to improvements given that anxiety was the most prevalent current concern reported by parents. - Effectiveness of parental strategies: Parents’ top endorsed strategies for anxiety management were comforting the child, providing reassurance, and encouraging the child to try new things. All of these strategies may be a helpful or poor fit depending on the context. It was noted that parents want a *quick fix*, which could relate to the anxiety management strategies parents are using, as the strategies parents endorsed tend to be effective in the moment but may not lead to long-term improvement in anxiety levels. It was recognized that long-term behavioral change without support is challenging for parents and leads to disengagement. HCS also noted difficulty closing the engagement gap given time pressures and an inability to easily link family with evidence-based resources.   Areas of discordance   - Did not identify key areas of concern: Anxiety was the most endorsed concern among parents (29%), but HCS perceived tantrums, sleep, toileting, and nutrition as the most prevalent unmet needs. |
| --- | --- |
| Behavioral challenges | Areas of confirmation   - Disruptive behaviors are a common concern: HCS reported that disruptive behavior problems were a common concern among parents. Parents frequently endorsed disruptive behaviors among the top three current/past concern, suggesting that parents need effective, actionable strategies. - Parents use ineffective strategies: HCS expressed concern that parents often engage in ineffective parenting strategies, and 33% of parents who reported concerns with disruptive behavior indicated that they had used spanking as a behavior management strategy. Yelling, repeating instructions, and physical restraint were also commonly endorsed.   Areas of expansion   - Various strategies to manage disruptive behavior were reported: Parents frequently endorsed yelling, repeating instructions, giving time-out, and offering incentive/rewards, while HCS reported that parents seek web-based resources, use strategies with little empirical basis (eg, weighted vests, CBD oil), rely on technology to distract children, and seek medication for developmentally typical challenging behavior. - Primary care contextual factors: Parents must continuously respond to children’s behavior, but with the typical 1-year interval of well-child visits, many parents end up responding to children’s aversive behaviors with coercion and punishment in the interim. When parents do visit their PCC, HCS often reported that parents wait too long to seek help. HCS reported that they can provide psychoeducational resources; however, HCS also admitted that they do not always know how and/or have the time to address parents’ concerns.   Areas of discordance   - HCS did not identify key areas of concern: HCS did not identify following directions and completing activities of daily living as common behavioral challenges, which were commonly identified as parental top concerns. |
| Nutrition and eating | Areas of confirmation   - Nutrition and eating is a common concern: 27% of parents identified nutrition and eating among the top three concern, and HCS often discussed nutrition and eating as a common concern. Both HCS and parents identified picky eating as a common challenge.   Areas of expansion   - Varying perceptions of strategy use: HCS indicated that parents tend to use inappropriate strategies to address nutrition and eating concerns (eg, allowing children to eat what they want). Parent responses indicated a mix of appropriate and inappropriate strategies. HCS frequently discussed that parents expect a *quick fix* for behavioral health concerns. Many of the inappropriate parental nutritional and eating strategies may produce an immediate positive response but fail to yield long-term improvements.   Areas of discordance   - Unaware of specific parental strategies: Parents commonly reported limiting high calorie drinks, maintaining a healthy weight, and punishing for not eating, but HCS did not discuss these strategies. - Addressing nutrition and eating in primary care: HCS indicated that they provide parental resources (eg, handouts and websites) or refer patients to specialists (eg, psychologist and nutritionist), but many parents reported that they had not discussed nutrition and eating concerns with their PCC (29%). Additionally, 17% of parents reported that they did talk to their PCC regarding nutrition and eating but did not find this helpful. |
| Parenting stress | Areas of confirmation   - Parenting stress is a common concern: Parents commonly endorsed parenting stress among their top three concerns (18%). HCS frequently noted that parents are frustrated with unworkable parenting strategies and observed that parents lack access to resources to manage parenting concerns.   Areas of expansion   - Effects of parental stress management strategies: Parents reported using distraction, normalization, opting out, overeating, and substance use to manage their stress. Participating in therapy/counseling was least endorsed by parents. Although HCS did not identify specific parental stress management strategies, HCS commented on parents’ tendency to *put off* getting help until their next office visit, which might be related to parenting stress and stress management strategies (eg, opting out). - Providers have narrow perceptions of parental stress: HCS were much more likely to use the term *frustration* rather than *stress* when discussing these parenting challenges, suggesting that HCS view their role as targeted toward specific issues parents have with their children. On the other hand, parent concerns about stress were broader and specific to themselves as opposed to their children (eg, self-care and dealing with their own emotions). - Perceptions of solutions: in focus groups: *Support* was often found in the same response as *frustration*, indicating that HCS recognize that parenting is difficult, frustration around behavior change is normal, and changing behaviors is a long-term process. Although HCS expressed that parents need support, they did not identify the specific types of support needed.   Areas of discordance   - Differing sources of stress identified: HCS believe that parents are stressed because of lack of knowledge of evidence-based strategies and because they expect a *quick fix* for their concerns. Parents, however, identified balancing self-care with other responsibilities, coping with difficult emotions/thoughts, and self-criticism as their main sources of stress. |
| Family communication | Areas of confirmation   - Lack of communication: Parents identified lack of communication as a top concern, and HCS expressed concern that parents delay care by waiting until office visits to communicate ongoing concerns with HCS and getting advice on parenting from other family members.   Areas of expansion   - Influences of family structure: Parents identified lack of open communication, effective coparenting strategies, and finding common ground with other caregivers as top problems. HCS recognized that family structure has an impact on parental needs and expressed concern that parents may not be able to get advice from other family members (eg, grandparents) because of family structure. - Difficulty finding common ground between parents and HCS: Parents frequently identified finding common ground with other caregivers as a top problem. HCS reported difficulty finding common ground with parents as they expressed that parents often reject advice from HCS, expect a quick fix, are misinformed, and do not understand that behavior change is a long process. HCS noted that they put much effort into trying to gather resources to better educate parents, although time and reimbursement constraints make this difficult to accomplish consistently.   Areas of discordance   - Did not identify key areas of concern: Family communication was endorsed as a top problem by 14% of parents, and parents identified toxic communication as a common concern. HCS did not explicitly discuss family communication concerns. - Differing perceptions of communication frequency: Parents commonly endorsed lack of communication as a top problem, whereas HCS often expressed the concern that parental communication efforts are overwhelming to the clinic (eg, large numbers of phone calls and patient electronic messaging), with parents requesting help with topics that were addressed in office visits. - Differing reports of strategy use: Parents endorsed several strategies to enhance communication including talking to their children about situations, providing rationales for expectations, and having positive conversations. HCS indicated that parents use social media or talk to other family members to address concerns. |
